# INVADEseq to identify cell-adherent or invasive bacteria and the associated host transcriptome at single-cell-level resolution

**DOI:** 10.1038/s41596-023-00888-7

**Published:** 2023-10-03

**Authors:** Jorge Luis Galeano Niño, Hanrui Wu, Kaitlyn D. LaCourse, Harini Srinivasan, Matthew Fitzgibbon, Samuel S. Minot, Cassie Sather, Christopher D. Johnston, Susan Bullman

**Affiliations:** 1 Human Biology Division, Fred Hutchinson Cancer Center, Seattle, WA, USA; 2 Bioinformatics Shared Resources, Fred Hutchinson Cancer Center, Seattle, WA, USA; 3 Data Core, Fred Hutchinson Cancer Center, Seattle, WA, USA; 4 Shared Resources Administration, Fred Hutchinson Cancer Center, Seattle, WA, USA; 5 Vaccine and Infectious Disease Division, Fred Hutchinson Cancer Center, Seattle, WA, USA

## Abstract

Single-cell RNA sequencing (scRNAseq) technologies have been beneficial in revealing and describing cellular heterogeneity within mammalian tissues, including solid tumors. However, many of these techniques apply poly(A) selection of RNA, and thus have primarily focused on determining the gene signatures of eukaryotic cellular components of the tumor microenvironment. Microbiome analysis has revealed the presence of microbial ecosystems, including bacteria and fungi, within human tumor tissues from major cancer types. Imaging data have revealed that intratumoral bacteria may be located within epithelial and immune cell types. However, as bacterial RNA typically lacks a poly(A) tail, standard scRNAseq approaches have limited ability to capture this microbial component of the tumor microenvironment. To overcome this, we describe the invasion–adhesion-directed expression sequencing (INVADEseq) approach, whereby we adapt 10x Genomics 5′ scRNAseq protocol by introducing a primer that targets a conserved region of the bacterial 16S ribosomal RNA gene in addition to the standard primer for eukaryotic poly(A) RNA selection. This ‘add-on’ approach enables the generation of eukaryotic and bacterial DNA libraries at eukaryotic single-cell level resolution, utilizing the 10x barcode to identify single cells with intracellular bacteria. The INVADEseq method takes 30 h to complete, including tissue processing, sequencing and computational analysis. As an output, INVADEseq has shown to be a reliable tool in human cancer cell lines and patient tumor specimens by detecting the proportion of human cells that harbor bacteria and the identities of human cells and intracellular bacteria, along with identifying host transcriptional programs that are modulated on the basis of associated bacteria.

## Introduction

In addition to malignant cells, the tumor microenvironment is composed of a range of different cell types including fibroblast, endothelial and varying immune cell types^1^. Furthermore, large-scale genomic studies have identified the presence of an intratumoral microbiota, including bacterial^2,3^ and fungal species^4,5^, across a range of human cancer types. Imaging data of patient tumors suggest that a portion of these intratumoral microbiota can be intracellular, located within immune and epithelial cell types^3,6,7^. By applying in situ spatial profiling to human oral and colorectal (CRC) cancers, our group recently demonstrated that intratumoral bacteria colonize tumor microniches that are less vascularized and are characterized by myeloid cell infiltration, T-cell exclusion and coincide with transformed cells with lower Ki67 levels and reduced wild-type p53 expression^8^. The development of single-cell RNA sequencing (scRNAseq) methods has provided unprecedented resolution of the varying transcriptional programs of eukaryotic cell types^9–11^ within tumor tissue. However, because these methods were developed for scRNAseq of eukaryotic cells, their RNA capture approaches rely on poly(A) selection of mRNA^12^ and, given that bacterial RNA typically lack a poly(A) tail^13^, such approaches have reduced ability to detect and identify intracellular or cell-associated bacteria. Here, we describe the invasion-adhesion–directed expression sequencing (INVADEseq)^8,14^ approach to facilitate the detection of bacterial RNA associated with eukaryotic single cells and to allow the analysis of host–bacterial interactions within patient specimens at the eukaryotic single-cell level.

### Development of the protocol

**Fig. 1 |:**
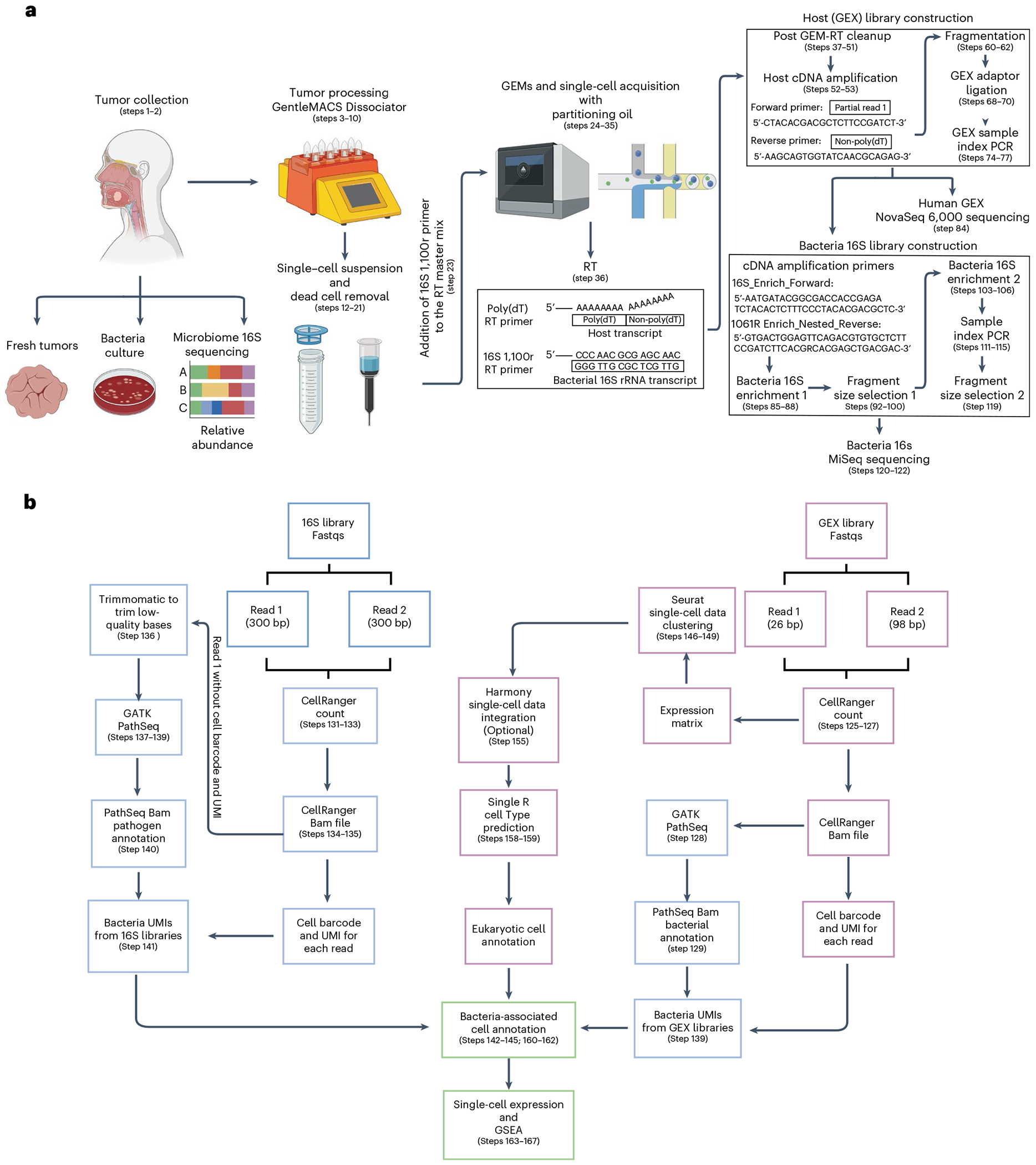
Tumor processing for single-cell RNAseq acquisition and computational pipeline for host and bacteria cell annotations, host-associated transcriptome and GSEA pathway enrichment analysis. **a**, Tumor samples were isolated from patients with gastrointestinal tract cancers. Bacteria culture in blood agars and microbiome 16S rRNA sequencing analysis were performed to screen tumor samples that were positive for bacteria. To obtain single-cell suspensions, tumor samples were processed using the gentleMACS-quality Octo Dissociator equipped with electrical heaters. The cell suspension was passed through a 70-μm cell strainer and dead cells were removed by magnetic sorting using LS columns. Single-cell suspensions were loaded onto a Chromium Chip K and processed with the 10x Chromium controller to capture single cells within a gel bead emulsion (GEM) containing a master mix with two primers, one that targets the polyadenylated host mRNA and second that targets the bacterial 16S rRNA gene. Following RT, the hosts (GEX) cDNA libraries were prepared and sequenced using the NovaSeq 600 platform. An aliquot from the GEX cDNA libraries were acquired to enriched for bacteria transcripts by amplifying the bacterial 16S rRNA gene. Using the BluePippin system fragment sizes between 955 and 1,215 bp were selected generating the bacteria 16S libraries that were sequenced using the MiSeq platform. **b**, Reads from the GEX libraries were mapped with the human reference genome GRCh38 using Cellranger Count. Then, the unmapped GEX reads with an adequate cell barcode and UMI count were processed via GATK PathSeq, thus obtaining bacterial UMI matrices for each bacteria-associated single cell. Reads from the 16S bacterial enrichment libraries were processed using Cellranger Count to obtain the corresponding barcode and UMI. Then R1 reads without a barcode or UMI were trimmed to remove low-quality bases and converted to BAM files to process through GATK PathSeq obtaining the bacteria UMI matrix for valid host cells from the GEX libraries. The bacterial UMI matrices from the GEX and 16S bacterial enrichment libraries were merged, removing UMI duplicates. Single-cell expression matrices from the GEX libraries were processed by Seurat followed by SingleR package software to obtain the annotations for each eukaryotic cell cluster. Harmony software was used to integrate single-cell datasets when it was required. The merged bacteria matrix was attached to the single-cell data identifying the host single cells that harbored bacterial transcripts. Gene expression profile and GSEA pathway enrichment analyses were performed based on the presence or absence of bacteria, at various taxonomic levels, at host single-cell-level resolution.

To identify the proportion of eukaryotic cells within the tumor microenvironment that harbor intracellular bacteria, the identity of both the host cell type and the associated bacteria, along with the host cell transcriptional profiles that are altered based on bacterial presence and transcriptional load, we have developed the INVADEseq approach^8^ (Fig. 1a,b). The INVADEseq approach builds on the backbone of the 10x Genomics Chromium 5′ scRNA assay, which utilizes the switching mechanism at the 5′ end of the RNA transcript (SMART) technology^1,2^. This includes a template-switching oligo (TSO) attached to the 10x barcode information on a bead within the Gel Bead-In Emulsions (GEMs), and the addition of the Moloney murine leukemia virus reverse transcriptase. In the standard protocol, an oligo(dT) primer is added to the GEMs (containing single cells), this oligo primes polyadenylated mRNA and the Moloney murine leukemia virus adds deoxycytidine to the 3′ end of the newly synthesized first-strand cDNA, which functions as an anchoring site for the TSO. This facilitates the reverse transcriptase to ‘switch’ template strands from the cellular RNA to the TSO and continue replication to the 5′ end of the TSO to generate cDNA that contains the 10x barcode information and unique molecular identifiers (UMIs). Importantly, as bacterial RNA transcripts typically lack poly(A) tails, standard approaches are not sufficient to detect cell-associated or intracellular bacteria. The INVADEseq approach takes advantage of this SMART technology and combines oligo(dT) (polyadenylated RNA) and conserved 16S rRNA gene (bacterial RNA) target primers in GEMs.

This novel yet simple approach will allow us to generate cDNA from both bacterial 16S rRNA and host mRNA transcripts. If this cDNA is generated in the same eukaryotic cell (one eukaryotic cell per GEM), bacterial cDNA and host cDNA will share an identical 10x barcode, but transcripts will have different UMIs.

### Applications of the method

**Fig. 2 |:**
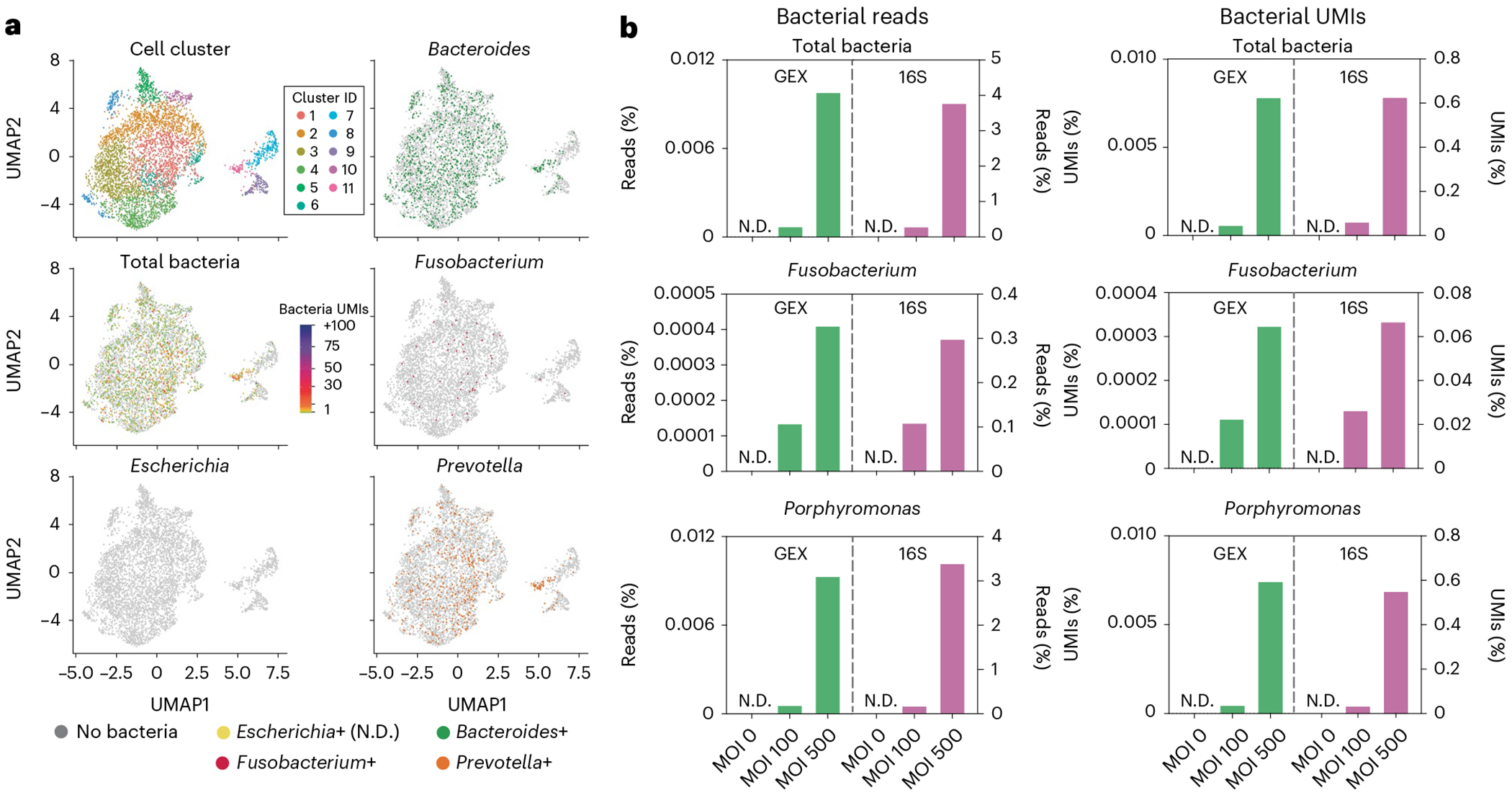
INVADEseq specificity and bacterial 16S rRNA gene enrichment libraries following cDNA amplification using a nested bacterial 16S primer. **a**, UMAP plots showing the eukaryotic cell clusters of epithelial cells derived from the CRC cell line HT29 incubated for 3 h with *Escherichia coli* DH5α *F. nucleatum* subsp. animalis COCA36, *Bacteroides fragilis* CTX25T, *Prevotella intermedia* 105CP, *Gemella haemolysans* CRC and *Veillonella parvula* CRC strain. Following the co-culture, the INVADEseq method detected host cells associated with bacterial species that had invasive properties. *E. coli* DH5α transcripts were not detected (N.D.) with single cells since this strain is nonadherent and noninvasive in these epithelial cells, affirming the specificity of the INVADEseq technique to detect invasive bacteria. Dataset taken from ref. 8. **b**, Epithelial cancer cells from the HCT-116 CRC cell line were incubated with *Fusobacterium nucleatum, Porphyromonas gingivalis* and *Prevotella intermedia* at an MOI of 100:1 and 500:1 for 3 h and processed with an uninfected control (MOI 0) for INVADEseq. Bar plots showing the proportion of bacterial reads (left) or bacterial UMIs (right) before (GEX; left *y* axis) or after the bacterial 16S enrichment step (16S; right *y* axis) with amplification using a nested primer that targeted the bacterial 16S rRNA gene. *F. nucleatum, P. gingivalis* and *P. intermedia* reads were not detected (N.D) in the MOI 0 sample. The bacterial reads accounted for 0.0007% and 0.0099% of total sequencing reads from the GEX library at an MOI of 100 and 500, respectively. Following the bacterial 16S rRNA gene enrichment step from the amplified cDNA, bacterial reads accounted for 0.2896% and 3.7787% of total sequencing reads at an MOI of 100 and 500, respectively. This represents an increase in the proportion of bacterial reads relative to human reads by three orders of magnitude following the bacterial 16S rRNA gene enrichment step. The percentage of UMIs or reads are relative to the total cell-positive GEM reads or UMIs obtained via the sequencing approach. These data demonstrate increased detection of host cell-associated bacterial reads and bacterial UMIs in the INVADEseq bacterial 16S rRNA gene libraries compared with the GEX libraries for total bacteria (*F. nucleatum, P. gingivalis* and *P. intermedia* combined), *Fusobacterium* and *Porphyromonas* transcripts at MOI 100 and MOI 500, as indicated. Each bar represents a single data point from the respective sample. The data used to generate this figure have been published previously^8^.

The INVADEseq method has been applied to identify cell-associated (adherent and invasive) bacteria (Fig. 2a), the specific eukaryotic cell types they associate with and their impact on host gene expression within human cell lines and oral squamous cell carcinoma (OSCC) tumor tissue^8^. This approach may be applied to identify host cell-associated bacteria and their relative transcriptional load, the host cell types that bacteria are associated with and to identify specific host cell transcriptional profiles altered by adherent or intracellular bacteria within bodily fluids or tissue specimens.

While this approach was applied to OSCC, it can also be applied to identify and profile such host–bacterial interactions in any mammalian fluid or tissue specimen at the host single-cell level. For example, this approach may be valuable in identifying cell-associated bacteria across additional cancer types demonstrated to harbor intratumoral bacteria^15^, or to enhance our understanding of host–bacterial interactions in a range of human diseases such as inflammatory bowel disease or cystic fibrosis^16,17^. The INVADEseq approach can elucidate the host transcriptomic changes and signaling pathways that are associated with specific pathogens from different clinical contexts.

Although we use a primer targeting a conserved region of the bacterial 16S rRNA, this approach can be further adapted by altering the INVADEseq primer introduced to target and enrich RNA that may not be captured via the oligo(dT) primer. For example, introducing a primer that targets a conserved region of the fungal Internal Transcribed Spacer 1 or 2 region of ribosomal genes^18,19^ would facilitate adaptation of the INVADEseq approach to detect host cell-associated fungi. This adaptation may be of value given two recent back-to-back studies demonstrating the presence of fungi within human cancer types^4,5^. However, beyond detection of the microbiota, our approach of spiking in a targeted reverse primer in addition to the oligo(dT) primer at the GEM stage, followed by further amplification and sequencing of this primer targeted cDNA will be beneficial for the detection and analysis of low-abundance or non-poly(A) transcripts, and determining their association with specific single cells.

### Comparison with other methods

The development of scRNAseq techniques have been crucial to understand the intrinsic heterogeneity of different eukaryotic cellular components within complex microenvironments. The commercially available Chromium 3′ and 5′ scRNA kits from 10x Genomics and the SMART-Seq Single Cell Kits use oligo(dT) priming to generate cDNA directly from single cells, capturing polyadenylated mRNA^20^. However, stable bacterial RNAs typically lack a poly(A) tail^21^, as such poly(A)-selecting approaches are limited in their abilities to capture non-poly(A)-tailed bacterial RNAs, unless RNA capture is off target^20^. Potential off-target bacterial transcripts have been identified computationally in poly(A)-selected scRNAseq libraries^22^ in addition to poly(A)-selected bulk RNA sequencing data^6,15,23^ from patient tumors. As it is estimated that a bacterial cell contains >100-fold less RNA than typical eukaryotic cells, bacterial transcripts within eukaryotic cells are greatly outnumbered by host cell transcripts^24,25^. This raises potential challenges with sensitivity to detect intracellular bacterial reads when relying on ‘off-target’ reads from poly(A)-selected sequencing libraries, and highlights the need for a microbial read enrichment step.

Given that 80–95% of the bacterial transcriptome is either 16S or 23S rRNA^26^ and that the 16S rRNA gene contains conserved regions (allowing universal bacterial targeting and amplification) that are flanked by variable regions (which facilitate taxonomic identification), we prime a conserved region of the 16S rRNA transcripts to generate bacterial cDNA with the INVADEseq approach. The INVADEseq approach, built on the backbone of the 5′ Chromium scRNA assay, primes both bacterial 16S rRNA and host mRNA simultaneously within host single-cell GEMs. Other approaches consist of measuring the relative abundance of microorganisms in tumor tissues by amplifying and sequencing the 16S rRNA gene in bulk. Although these techniques can taxonomically resolve the microbes that reside in the bulk tissue, they cannot distinguish the host cellular compartments that such microorganism are interacting with, and the corresponding host-associated transcriptome. Conventional gentamicin protection assays that measure the capacity of bacteria to invade host cells can only estimate the number of internalized bacteria overall. However, bacterial internalization is a nonsynchronous process, meaning that not all bacterial cells have the same capacity to invade the host cells. Furthermore, there is also functional heterogeneity in the susceptibility of host cells to being infected. This functional variability makes it difficult to calculate exactly the absolute number of host cells that are being infected by a pathogen using the standard approaches. Our INVADEseq method overcomes this issue by measuring the number of bacterial UMI transcripts for each host cell. Some of them can harbor more than 100 bacterial transcripts, whereas others harbor only one or no bacteria transcripts (Fig. 2a).

The approach is designed as an ‘add-on’ step to the standard 5′ scRNAseq approach. In addition to the standard 5′ Chromium scRNA gene expression (GEX) library preparation, a portion of the host bacterial cDNA is processed to generate the INVADEseq 16S bacterial enrichment library, which involves bacterial read enrichment through a nested amplification step targeting the 16S rRNA cDNA (Fig. 2b). Our downstream INVADEseq computational analysis includes the identification of potential ‘off-target’ microbial reads detected through the standard 10x Genomics GEX libraries, in addition to the bacterial reads enriched in the additional INVADEseq 16S bacterial enrichment library (Fig. 1b). The 10x barcode, which is retained in the GEX and INVADEseq 16S bacterial enrichment libraries, facilitates the identification of host single cells with cell-adherent or intracellular bacteria.

### Expertise needed to implement the protocol

The INVADEseq protocol requires expertise in the following: aseptic techniques to prevent bacterial contamination or cross-contamination between specimens or samples; cell culture and tissue processing, single-cell isolation and handling, and molecular biology techniques; next generation library preparation and sequencing; and data analysis. A bioinformatician is necessary for processing the data and running the pipeline to taxonomically resolve the microorganisms that are associated with host single cells, annotate the host–cell clusters and exclude low-quality cells or cell-free GEMs from downstream analysis. Experience in the microbiome and mammalian transcriptome is needed to interpret the data.

### Limitations

The protocol is in accordance with the standard 10x Genomics 5′ scRNA seq approach to maximize mammalian cell viability and we do not modify cell lysis steps to enhance lysis of Gram-negative or Gram-positive bacterial cells. Identification of neutrophils has been challenging since they exhibit low RNA content and high levels of RNase and other inhibitory compounds, resulting in fewer transcripts detected in the GEX libraries. In some cases, the acquisition of a limited number of high-quality single cells can reduce the ability to generate well-defined cell clusters, thus restricting the analysis approach when comparing total bacteria-positive versus total bacteria-negative single cells in the entire sample. Furthermore, the application of INVADEseq to specimens with a very low bacterial biomass may be challenging due to the number of bacteria-associated cells identified and the possibility of low bacterial transcriptional load or UMI counts. In these cases, a bacterial UMI of one may be the only bacterial UMI threshold possible for identifying bacteria-associated single cells and for comparative data analysis. However, if the UMI threshold needs to be reduced to one, this suggests the bacterial biomass is very low and the user should pay particular attention to the bacterial taxa identified due to the potential impact of contamination from reagents used during sample processing^27,28^. Similar to all microbiome studies, it is important to assess whether the bacterial taxa detected make biological sense in the context of the tissue type or disease state being analyzed. For example, in bulk RNA and DNA sequencing approaches, groups have applied in silico contamination prediction methods, using a curated list of common bacterial contaminants, to limit the impact of contaminants on samples with low bacterial biomass^29,30^. To maximize the successful application of the INVADEseq approach to a particular specimen or disease type with unknown microbial load, confirmation of intracellular or cell-associated bacteria via RNAscope imaging with a eubacterial probe is recommended^8^. If intracellular or cell-associated bacteria cannot be detected through imaging approaches, it will be challenging to detect cell-associated bacteria via INVADEseq.

In patient specimens, we have previously noted the localization of bacteria within necrotic regions of the tissue and association with cells with lower proliferation rates, both factors may result in reduced cell viability measurements and challenges capturing sufficient viable single cells from bacteria-positive tumor specimens.

Additionally, the INVADEseq technique is designed to taxonomically resolve viable bacteria through the introduction of a 16S rRNA gene targeting primer, and therefore it cannot analyze the entire bacterial transcriptome. This approach will facilitate the identification of bacterial taxa associated with mammalian cells, the identity of the mammalian cells and the altered transcriptome of the mammalian cell but not the associated bacterial transcriptome.

When the INVADEseq protocol was performed in OSCC tumors, the majority of the cell associated bacteria were Gram-negative; however, we did detect Gram-positive taxa, including *Parvimonas micra*, through this approach. Gram-positive bacterial cells and *Mycobacteria* are more difficult to lyse compared with Gram-negative organisms, and it is possible that the standard 10x Genomics 5′ scRNAseq approach lysis may not be sufficient for lyses of these organisms. In cases where Gram-positive taxa and *Mycobacteria* are of particular interest, groups may want to introduce additional lysis approaches post-capture of single cells in the GEMs. However, the impact of such additional lysis steps on the integrity of mammalian RNA needs to be evaluated.

We have confirmed the selectivity of the INVADEseq approach to detected cell-associated bacteria and show an increased detection rate of cell-associated bacteria with increasing infection rate^8^. However, the exact level of sensitivity of the INVADEseq approach is challenging to assess due to variability in cell adhesion and invasion dynamics in co-cultured bacteria and eukaryotic cellular experiments. Similar to standard mammalian single-cell sequencing regarding the detection of rare cell populations and rare cell transcripts, the sensitivity of INVADEseq for detecting bacteria-associated single cells is dependent on the total number of single cells captured and the number of sequencing reads obtained.

### Experimental design

#### Validation of the method

In previous work, we have demonstrated that the introduction of the bacterial 16S rRNA primer did not substantially alter the gene expression profile of CRC cancer cells^8^. Additionally, to validate this approach, we co-cultured colon cancer cell lines with a range of different bacterial species, including cell adherent and invasive *Fusobacterium nucleatum* and non-cell-adherent *Escherichia coli* DH5α for 3 h before performing the INVADEseq approach. In this validation experiment, the INVADEseq method detected cell-adherent and invasive bacterial taxa but did not detect cell associated transcripts from the nonadherent and noninvasive bacterium *Escherichia coli* DH5α, supporting selectivity of the approach^8^ (Fig. 2a). Additionally, we have assessed this approach on the HCT116 colon cancer cell line with different multiplicities of infection (MOIs) of specific bacterial taxa (MOI 0, MOI 100 and MOI 500), and confirmed a dose-dependent increase in the detection of cell-associated bacteria, along with alterations in the transcriptional signatures of host cells (Fig. 2b). As the input for the bacterial enrichment library is the amplified cDNA following both poly(A) selection of host mRNA and 16S RNA gene selection of bacterial RNA from host single cells, and we are not depleting host cDNA but rather enriching bacterial cDNA within the sample, the majority of cDNA postbacterial enrichment will still be host, we are simply enriching bacterial cDNA within this background (Fig. 2b). For example, in our validation analysis detailed in Fig. 2, the bacterial reads accounted for 0.0007% and 0.0099% of total sequencing reads from the GEX library at a bacterial MOI of 100 and 500, respectively. Following the bacterial 16S rRNA gene enrichment step from the amplified cDNA, bacterial reads accounted for 0.2896% and 3.7787% of total sequencing reads at a bacterial MOI of 100 and 500, respectively. This represents an increase in the percentage of bacterial reads relative to human reads by three orders of magnitude following the bacterial 16S rRNA gene enrichment step. Additionally, please note that bacterial cell adhesion and cell invasion capabilities can vary drastically between bacterial taxa even at the strain level, additionally these capabilities can vary by host cell type. The processing of the specimens and cells is in accordance with the standard 10x 5′ Chromium scRNA assay to maximize high-quality mammalian single cells rather than to enhance lysis of bacterial cells, because to detect cell-associated bacteria, we first need high-quality host single cells.

#### Controls

The control conditions will depend on the analysis approach that is implemented. For example, in our cell line and bacterial strain co-culture experiments, we included an uninfected cell line as a bacterial-free, negative control (Fig. 2b). For our cell line and bacterial strain co-culture experiments, we included bacterial taxa that we confirmed were cell invasive via confocal microscopy, and these were a positive control when validating our approach. However, within samples, bacteria-free cells may be used as a control group for comparative transcriptomics against bacteria-associated cells. For analysis applied to individual tumor specimens, we aimed to obtain a minimum of 4,000 single cells from each patient tumor to allow for comparison of bacteria-positive and bacteria-negative cells within the same cell cluster or cell type. If the number of single cells captured is limited and there are low numbers of distinct cell clusters, then an ‘all cluster’ analysis can be performed comparing the host transcriptome between total bacteria-positive versus total bacteria-negative single cells in the whole sample. In individual patient specimens where we generated sufficient single cells (>4,000 single cells) and identified bacteria, we performed intratumoral analysis of single cells based on the presence or absence of cell-associated bacteria. Additionally, we have integrated single-cell data from several patients with the same cancer type to identify transcriptional profiles altered in specific cell types by general bacteria (total bacteria) and specific enriched bacterial taxa such as *Fusobacterium* and *Treponema* species. Furthermore, by increasing the bacterial UMI threshold to identify bacteria-associated single cells, the number of differentially expressed genes increased when comparing bacteria-positive and bacteria-negative cells, probably reflecting impacts of higher bacterial load or transcriptional levels within cells. Although the detection of a single bacterial UMI that shares a 10x barcode with a host single cell can be considered a bacterial-associated cell, the UMI metric may act as a proxy for bacterial transcriptional load, so an increased UMI threshold for bacteria may be applied to identify highly impacted transcriptional pathways. Additionally, in our previous analysis where we applied a UMI metric cutoff of ≥3, single cells harboring bacterial UMIs ≤3 were not included in the comparison group, only true bacteria-negative (bacteria UMI 0) single cells^8^.

## Materials

### Biological materials

- Fresh tumor specimens isolated from patients with OSCC were processed for INVADEseq
  ▴ **CAUTION** Any experiments using human material should adequately fulfill the institutional and national regulations; therefore, informed consent must be obtained.
- The human colon cancer epithelial cells lines HT-29 (RRID: CVCL_0320, ATCC) and HCT 116 (RRID: CVCL_0291, ATCC) were cultured in McCoy’s 5A with l-glutamine (Corning) and penicillin–streptomycin and supplemented with 10% (vol/vol) FBS (Sigma). Penicillin–streptomycin was not used in cell lines when co-cultured with or without bacteria for INVADEseq. Cell cultures were incubated for 3 h at 37 °C in 5% CO_2_
  ▴ **CAUTION** It is recommended that the cell lines be regularly tested to ensure that they are not misidentified or cross-contaminated. Additionally, it is important that they be regularly tested for *Mycoplasma* contamination.
- Bacterial strains *Escherichia coli* DH5α (ThermoFisher Scientific), *F. nucleatum* subsp. animalis COCA36 (Bullman Lab strain), *Bacteroides fragilis* CTX25T (Bullman Lab strain), *Prevotella intermedia* 105CP (Bullman Lab strain), *Gemella haemolysans* CRC (Bullman Lab strain) and *Veillonella parvula* CRC (Bullman Lab strain) were cultured from cryostocks on fastidious anaerobe agar plates (Grainger, Neogen) supplemented with 10% defibrinated horse blood (Lampire Biological Laboratories, Fisher). Bullman Lab strains are in-house bacterial strains isolated from patient CRC tumors. Bacterial culturing occurred under anaerobic conditions using the anaerobic chamber Anaerobe Systems AS-580 and incubated at 37 °C for 24–48 h, depending on the bacterial strain
  ▴ **CAUTION** As INVADEseq is designed to identify bacteria that are cell associated, including highly cell invasive taxa, many of these organisms are considered human pathogens and potentially contribute to the development of cancers. Therefore, this protocol must be performed in laboratories that follow at least biosafety level 2 precautions.

### Reagents

- McCoy’s 5A (Modified) medium (Thermo Fisher Scientific, cat. no. 16600082)
- Gibco l-glutamine 200 mM (Thermo Fisher Scientific, cat. no. 25030081)
- FBS (Sigma-Aldrich, cat. no. F4135)
- Penicillin–streptomycin (10,000 U/mL) (Thermo Fisher Scientific, cat. no. 15140122)
- Defibrinated horse blood (Lampire Biological Laboratories, cat. no. 7233401)
- ACUMEDIA Fastidious Anaerobe Agar (W.W. Grainger, cat. no. 39M684)
- Trypsin–ethylenediaminetetraacetic acid (0.25%), phenol red (Thermo Fisher Scientific, cat. no. 25200056)
- Tumor dissociation kit (human) (MiltenyiBiotec, cat. no. 130-095-929)
- Red blood cell lysis solution (10×) (MiltenyiBiotec, cat. no. 130-094-183)
- MACS SmartStrainers 70 μm (MiltenyiBiotec, cat. no. 130-098-462)
- Phosphate-buffered saline, pH 7.4 (Thermo Fisher Scientific, cat. no. 10010023)
- UltraPure BSA (invitrogen, cat. no. AM2616)
- Dead cell removal kit (MiltenyiBiotec, cat. no. 130-090-101)
- LS columns (MiltenyiBiotec, cat. no. 130-042-401)
- Chromium Next GEM Chip K Single Cell kit (10x Genomics, cat. no. PN-1000287)
- Chromium Next GEM Single Cell 5′ reagent kits v1 and v2 (10x Genomics, cat. no. PN-1000165 and PN-1000263, respectively)
- Chromium Single Cell 5′ library kit, (10x Genomics, cat. no. PN-1000002)
- Dual index kit TT Set A, 96 reactions (10x Genomics, cat. no. PN-1000215)
- Buffer EB (Qiagen, cat. no.19086)
- 10% Tween 20 nonionic detergent (Bio-Rad cat. no. 1610781)
  ▴ **CRITICAL** The INVADEseq protocol was developed using the commercial kits for the Chromium Next GEM Single Cell 5′ platform (Steps 23–84). Alternative scRNAseq systems should be validated and optimized for the detection of bacteria-associated host cells adapting the corresponding manufacturer’s protocol. This detailed protocol is relating to the Chromium Next GEM Single Cell 5′ v1 kit; all steps are comparable for the Chromium Next GEM Single Cell 5′ v2 kit with the inclusion of Lucigen’s MasterAmp 10× PCR Enhance (product number ME81210) to replace the cDNA Additive reagent during ‘Generation of INVADEseq bacterial 16S rRNA gene libraries’ detailed in the methods section below.
- Bacteria targeting primer added to the RT mix (Step 23): 16S 1100R (5′ GGGTTGCGCTCGTTG 3′)
- Customized bacterial 16S RNA primers for enrichment of bacterial cDNA (Steps 85 and 103): 16S Enrich Forward (5’AATGATACGGCGACCACCGAGATCTACACTCTTTCCCTACACGACGCTC3′) and 1061R Enrich Nested Reverse (5′GTGACTGGAGTTCAGACGTGTGCTCTTCCGATCTTCACGRCACGAGCTGACGAC3′)
  All custom primers were obtained from Integrated DNA Technologies).
  ▴ **CRITICAL** The customized bacterial 16S rRNA primers for INVADEseq were tested in house and are applicable for users interested in assessing cell associated bacteria via the INVADEseq approach as described in this protocol. However, researchers can design their own primers targeting a particular bacterial gene of interest or an alternative region of the 16S rRNA or 23S rRNA genes. The custom primer added to the RT mix would need to be a reverse primer (similar to 16S 1100R). For the enrichment step (similar to enrichment of bacterial cDNA, above), the forward primer should remain the same as the 16S Enrich Forward (which aligns to the Illumina adaptor and facilitates retention of the 10x barcode and UMI) and the reverse primer should be a nested primer (similar to 1061R Enrich Nested Reverse) relative to the custom primer added to the RT mix and should contain the following Illumina adapter sequence at the 5′ region GTGACTGGAGTTCAGACGTGTGCTCTTCCGATCT (similar to 1061R Enrich_Nested_Reverse) to facilitate Illumina library preparation. Bacterial rRNA genes are a suitable target due to their high contribution to the bacterial transcriptome and the presence of conserved regions for broad bacterial targeting, flanked by variable regions for downstream taxa identification via analysis of resulting sequencing data.
- Agilent high-sensitivity DNA kit (Agilent Technologies, cat. no. 5067-4626)
- Nuclease-free water (Thermo Fisher Scientific, cat. no. AM9937)
- Glycerol, 99.0%, molecular biology grade, ultrapure (Thermo Scientific, cat. no.J16374.K2)
- Ethanol, pure (200 proof, anhydrous) (Millipore Sigma, cat. no. E7023-500ML)
- SPRIselect reagent kit (Beckman Coulter, cat. no. B23318)
- 10% Tween 20 nonionic detergent (Bio-Rad, cat. no. 1662404)
- Qiagen Buffer EB (Qiagen, cat. no. 19086)
- BluePippin 1.5% agarose cassettes (Sage Sciences, cat. no. HCT1510)
  ▴ **CAUTION** Agarose gels can cause irritation to the eyes, mouth, skin and upper respiratory tract. The monomer acrylamide is a probable human carcinogen and may also cause adverse reproductive and nervous system health effects. Use adequate personal protective equipment when handling these reagents.
  ▴ **CRITICAL** Sterility of reagents used for specimen processing is essential for the accurate interpretation of downstream microbial data generated from the INVADEseq approach.

### Equipment

- Countess II FL automated cell counter (Thermo Fisher Scientific, cat. no. AMQAF1000)
- GentleMACS Octo Dissociator (MiltenyiBiotec, cat. no. 130-095-937)
- Tube Revolver Rotator (Thermo Fisher Scientific, cat. no. 88881001)
- Chromium 10x controller (10x Genomics, cat. no. PN-1000204)
  ▴ **CRITICAL** The INVADEseq approach should be compatible with any of the 10x Genomics Chromium platforms that support the 5′ scRNAseq assay.
- MidiMACS Separator (MiltenyiBiotec, cat. no. 130-042-302)
- MiSeq Sequencing system (Illumina, cat. no. SY-410-1003)
- NovaSeq 6000 Sequencing system (Illumina, cat. no. 20012850)
- NextSeq 1000 and NextSeq 2000 Sequencing systems (Illumina)
- C1000 Touch Thermal Cycler with 96-Deep Well Reaction Module (Bio-Rad, cat. no. 1851197)
- Agilent 4200 TapeStation System (Agilent cat. no. G2991BA)
- 64-bit high-performance computing cluster node running Ubuntu 18.04; Intel Gold 6154 central processing unit with 36 cores; 768 GB of random access memory; required installed and configured software and packages
- BluePippin platform (Sage Sciences, cat. no. BLU0001)

### Software

- GATK PathSeq V4.1.3.0 Pathogen discovery pipeline (Broad Institute)
- Seurat v4.0.4 Tools for Single Cell Genomics (Satija Lab, New York Genome Center)
- Harmony, an R package for single-cell integration (Center for Data Sciences, Brigham and Women’s Hospital^31^)
- SingleR v1.4.1 an R package for Single-cell Recognition (Division of Pulmonary, Critical Care, Allergy, and Sleep Medicine, Department of Medicine, University of California, San Francisco^32^)
- CellRanger v6.1.1 to perform cell clustering and secondary analysis (10x Genomics)
- Nextflow 20.09.0 (Centre for Genomic Regulation, The Barcelona Institute for Science and Technology)
- BEDTools 2.29.2 (Department of Biochemistry and Molecular Genetics, University of Virginia, Charlottesville, VA)
- SAMtools 1.10 (Wellcome Trust Sanger Institute)
- Trimmomatic 0.39 to remove low-quality bases from fastq files (Institute of Bio- and Geosciences: Plant Sciences, Forschungszentrum Jülich)
- ClusterProfiler 4.1.1 to perform Gene Set Enrichment Analysis (GSEA) (Yu lab, Southern Medical University, Guangdong, China)
- R 4.2.0 R Foundation for Statistical Computing
- Python 3.7 Python Software Foundation
- PySam 0.20.0 (Andreas Heger and Kevin Jacobs)
- Pandas 1.3 AQR Capital Management

## Reagent setup

### Enzyme preparation for tumor dissociation

Reconstitute lyophilized enzyme H by adding 3 ml of RPMI 1640 or Dulbecco’s modified Eagle medium (DMEM) culture media into the vial. Reconstitute enzyme R by adding 2.7 ml of RPMI 1640 or DMEM culture media. Reconstitute enzyme A by adding 1 ml of Buffer A, which is supplied by the human tumor dissociation kit. For each enzyme, prepare several aliquots of appropriate volumes to avoid continued cycles of freezing and thawing. Store aliquots at −20 °C and the soluble enzymes are stable for a period of 6 months.

### Gel beads preparation

Use one tube of gel beads per sample. Equilibrate the gel beads strip at room temperature (24 °C) 30 min before use on the Chromium 10x controller. Unused gel beads can be stored at −80 °C for 3–6 months avoiding repeated freeze–thaw cycles. To vortex the beads, attach the 10x Vortex Adapter to the top of standard laboratory vortexes. Following vortexing the beads for 30 s, remove the gel bead strip form the adapter and centrifuge briefly for −5 s. Confirm that there are no bubbles at the bottom of the tubes and the volume levels are even. Place the gel bead strip back into the holder.

### 50% glycerol solution

Mix an equal volume of 99% glycerol, molecular biology grade and filtered through a 0.2-μm filter. Make several aliquots of adequate volumes. Store the aliquots at −20 °C for up to 6 months. Glycerol solution should be calibrated at room temperature before use.

### 10x magnetic separator

The 10x magnetic separator comes with two positions of the magnet referred as high and low relative to the orientation of the tube. Flip the magnetic separator over to shift between high (magnet•High) or low (magnet•Low) sides.

### Enzymatic fragmentation

Ensure that the enzymatic fragmentation reaction is prepared on ice and then loaded into a thermocycler previously precooled to 4 °C before starting the fragmentation, end repair and A-tailing incubation steps (Step 61).

### SPRIselect reagent handling

Pipette calibration and accuracy is particularly important when handling with the SPRIselect reagent. After taking the desired volume, examine the pipette tips to confirm the correct volume before it is transferred to the mix. Mix thoroughly by pipetting since insufficient mixing can lead to inconsistent results. For washing the SPRIselect beads, prepare fresh solutions of 80% ethanol in advance.

### Dynabeads MyOne SILANE preparation

Vortex thoroughly (≥30 s) immediately before adding to the mix. To ensure that the beads are not settled in the bottom of the tube, take the full liquid volume with a pipette tip and visually confirm that the solution is homogeneous. If clumps are still present, resuspend the vial by pipetting. Do not centrifuge the vial before using it.

### Bacteria 16S rRNA primer reconstitution

Resuspend all lyophilized primers with nuclease-free water to a concentration of 100 μM and store at −20 °C for up to 1 year.

## Equipment setup

### GentleMACS Octo Dissociator

The gentleMACS Octo Dissociator comes with a variety of predefined programs to process different type of tissue. Before using this equipment ensure to select the appropriate program to dissociate or homogenize the tissue of interest. For tissue processing using enzymes, ensure to attach the heater to the corresponding tube position. Depending on the constitution of the tumor tissue, it is recommended that the sample be split into smaller pieces before processing.

### C1000 Touch Thermal Cycler

For reverse transcription (RT), cDNA amplification, fragmentation and ligation, it is recommended that the steps, temperatures, duration and number of cycles be set up in advance for each thermocycler reaction during this protocol. From the touchscreen display, researchers can add new protocols indicating the temperature and duration for each step. The GOTO function instructs the thermocycler to repeat a set of steps in a loop. It is also important to introduce the volume in μl and the lid temperature for each reaction from the INVADEseq method. It is also advisable to save the settings in advance for each thermocycler reaction during the procedure.

### Chromium 10x controller

Assemble and load the Next GEM Chip K into the chip holder. The assembled chip holder should stay flat to the bench top with the lid closed. Following loading the Chip K rows labeled as 1, 2 and 3 with the respective solutions (Step 25), hook the 10x gasket on the left- and right-hand tabs of the chip holder. Ensure the 10x gasket holes are aligned with the wells from the chip. Press the eject button from the touchscreen and place the chip holder in the Chromium 10x controller tray. Press the button again on the touchscreen to retract the tray and run the standard program that it is displayed on the screen. At the end of the run (~18 min), the Chromium-quality Controller will beep. Press the button to eject and empty tray to continue the protocol.

### BluePippin platform

The BluePippin optical system must be calibrated before every run. The platform is provided with a calibration fixture that can be placed over the light-emitting diode (LED) detector on the optical nest. To begin, press the ‘Calibrate’ button to open the ‘LED calibration’ window, place the calibration fixture in the optical nest and ensure that all five LED detectors are covered. Then, close the lid and from the ‘LED calibration’ window press the ‘Calibrate’ button to perform the calibration run. Once calibration is successful, the ‘Calibration Status’ field will contain the message ‘Calibration OK’.

### NovaSeq 6000 sequencing system

Submit libraries for sequencing on one lane of an Illumina HiSeq 4000 instrument (paired-end, 75 bp reads) according to the manufacturer’s directions. We typically aim for an average depth of 1 million reads per single cell to capture low-expressing genes and to enhance the detection of rare cell populations from population in the tumor tissue. Alternative sequencing options to achieve comparable sequencing depth are the NextSeq 550 with high-output flow cell or NovaSeq 6000 SP flow cell on one lane of an XP workflow.

### MiSeq sequencing system

Bacterial 16S cDNA libraries were sequenced on a MiSeq Illumina sequencer, establishing a paired-end 300 base read length (PE300) using V3 reagents and multiplexing between seven and nine samples per flow cell. Secondary analysis on this instrument was performed by using MiSeq Reporter Software v2.5.1, monitoring base calling and quality scores by real-time analysis (RTA) v1.18.54 (Illumina).

▴ **CRITICAL** Similar to the standard GEX sequencing approaches, increasing the number of reads per cell can increase the probability of capturing transcripts of low abundance, since bacterial transcripts are a minor component of the total human transcripts, increasing the number of reads can assist in detecting bacterial transcripts.

## Procedure

### Tumor dissociation for single-cell generation

#### • TIMING ~2 h

1. Immerse freshly biopsied or resected tumors in precooled RPMI culture media without antibiotics and transport at 4 °C until tissue processing in the laboratory.
  ▴ **CRITICAL STEP** It is essential that the tissue is processed as fast as possible to maintain cell viability. The tissue should be processed as soon as possible, ideally beginning within an hour following biopsy or resection to preserve RNA transcripts and prevent the ex vivo loss of strict anaerobic bacteria and overgrowth of aerobic bacteria.
  ◆ TROUBLESHOOTING
2. Dissect each tumor into small pieces ~2–4 mm in diameter using sterile disposable scalpels.
3. Load the tumor pieces into a gentleMACS C tube, which contains a stator and a rotor element that provide the mechanical forces to extract cells from the tissue.
4. Add 325 μl of the mix of enzymes (enzymes H, R and A), provided by the Tumor Dissociation Kit, to the C tube as follows: 200 μl of enzyme H, 100 μl of enzyme R and 25 μl of enzyme A resuspended in 4.7 ml of RPMI 1640 or DMEM.
  ▴ **CRITICAL STEP** The enzyme volumes are calculated based on the size of the tumors. For tumors below 0.2 g, prepare the following mix of enzymes: 100 μl of enzyme H, 50 μl of enzyme R and 12.5 μl of enzyme A resuspended in 2.2 ml of RPMI 1640 or DMEM.
  ▴ **CRITICAL STEP** To avoid the expression of immediate-early genes associated with cellular stress originated by tissue processing, the addition of 45 μM actinomycin D (for 35 °C digestion) has been demonstrated to inhibit transcriptomic artifacts^33^.
  ◆ TROUBLESHOOTING
5. Tightly close the C tube and attach it upside down onto the tissue dissociator and lock in with the clamp. Make sure that the tumor pieces are in contact with the rotor.
  ▴ **CRITICAL STEP** For enzymatic and mechanical tumor dissociation, the instrument is equipped with heaters for each of the eight tube positions. By pressing the clamps, the heaters can be attached to each individual tube positions, thus linking the heaters to the instrument using its electrical contact pins.
6. Select the appropriate gentleMACS Program for your tumor tissue type; for OSCC tumors use ‘37C_h_TDK_3’, designated for tough tumor tissue.
  ▴ **CRITICAL STEP** Change the program settings of the gentleMACS Octo Dissociator to 37C_h_TDK_1 or 37C_h_TDK_2 for soft and medium tissues, respectively, thus reducing tissue damage.
  ◆ TROUBLESHOOTING
7. After termination of the program, detach the C tube from the gentleMACS Dissociator.
  ▴ **CRITICAL STEP** Use gentle pipetting and mixing when handling the cells after dissociation.
8. Centrifuge the cell suspension at 300*g* for 30 s at 4 °C to ensure all sample is removed from the rotor and pooled at the bottom of the C tube.
9. Add 10 ml of RPMI 1640 or DMEM to the dissociated tissue and transfer the cell suspension into a pre-wetted 70-μm pore-size strainer with culture media to remove clumps of cells and tissue debris. Wash the strainer with 10 ml of additional RPMI 1640 or DMEM.
10. Centrifuge the cell suspension at 300*g* for 7 min and aspirate the supernatant.
11. Resuspend the pellet of cells with 1 ml of chilled and freshly prepared 1× red blood cell lysis solution (MiltenyiBiotec) and incubate for 10 min at 4 °C.
12. Wash the cells with 10 ml of DPBS + 0.04% ultrapure BSA and pellet the cells at 300*g* for 10 min. Resuspend the cells in 150 μl of DPBS + 0.04% BSA and proceed to measure the cell count and viability using a trypan blue exclusion assay or your preferred method using 10 μl of this concentrated suspension.
13. Resuspend the cell pellet in 1 ml of 1× binding buffer containing 100 μl of the Dead Cell Removal MicroBeads. Mix well and incubate for 15 min at room temperature.
  ▴ **CRITICAL STEP** Dead Cell Removal MicroBeads are susceptible to bacterial contamination; take care to handle under sterile conditions. Handle the Dead Cell Removal MicroBeads with wide-bore pipette tips. Choose an appropriate MACS Column and MACS Separator according to the number of total cells. Always wait until the column reservoir is empty before proceeding to the next step.
  ▴ **CRITICAL STEP** If the sample contains greater than 1 × 10^7^ cells, use 150 μl of beads.
14. Place the LS columns in the magnetic field of the MACS Separator.
15. Wash the columns by adding 3 ml of cool (4 °C) 1× binding buffer and discard the flow through.
16. Add the cell suspension on the top of the column. Collect the flow through below in a 50 ml tube. This fraction contains the live cells.
17. Wash the LS column four times by adding 3 ml of cool (4 °C) 1× binding buffer and collect the flow through into the same 50 ml tube containing the live cells. Wait for each wash to finish before beginning the subsequent wash.
18. Remove and discard the column from the magnetic separator. Centrifuge the 50 ml tube at 300*g* for 10 min at 4 °C.
19. Discard the supernatant and resuspend in 1 ml of PBS + 0.4% BSA and transfer cells to a 1.5 ml Lo-bind eppi tube.
20. Wash cells twice with 1 ml of PBS + 0.4% BSA, centrifuging at 300*g* for 5 min at room temperature each time, and removing and discarding the supernatant by pipetting.
21. Resuspend the final cell pellet in 150 μl of PBS + 0.4% BSA and measure the cell count and viability using the trypan blue exclusion assay measured by a Countess III FL cell counter.
  ▴ **CRITICAL STEP** Dead cell removal can be performed twice if cell viability is below 75%. To ensure a single-cell suspension, it is recommended that the sample be passed through 70-μm cell strainers to remove cell clumps.
  ◆ TROUBLESHOOTING
22. Prepare 100 μl of cells in PBS at a concentration of 700–1,200 cells/μl to load onto the 10x Chromium controller. Store cells on ice until they are loaded. This may require dilution; always re-assess the cell count and viability after preparing a dilution.
  ∎ **PAUSE POINT** Store excess cells at −80 °C in an appropriate cryoprotectant for up to 6 months.
  ▴ **CRITICAL STEP** The dead cell removal protocol can be repeated twice to increase the percentages of viable cells if it is required.

### Single-cell acquisition for RNA sequencing

#### • TIMING ~4 h

- Prepare the master mix as follows:

**Table:** 

Component	Amount (μl)	Final concentration
RT reagent B PN-2000165^a^	18.8	1×
Poly(dT) RT primer PN-2000007^a^	7.3	1×
Reducing agent B PN-2000087^a^	1.9	1×
RT enzyme C PN-2000085^a^	8.3	1×
16S primer 1100R (100 μM)	6	14.2 μM
Total	42.3	

a Provided by the Chromium Next GEM Single Cell 5’ Reagent kits v2 (10x Genomics, cat. no. PN-1000263)
  ▴ **CAUTION** It is important to perform this assay in an RNase-free environment to avoid RNA degradation. This includes the use of sterile RNase-free barrier pipette tips and certified Rnase- and DNase-free microfuge tubes. Wear gloves when handling RNA and all reagents, as skin is a common source of RNases. All solutions should be made with sterile RNase-free water and used only for RNA work.
- For each tumor sample, add 36.3 μl master mix into a tube from an eight-tube PCR strip on ice.
- Prepare the Chromium Next GEM Chip K by adding 50% glycerol into the wells of the chip as follows: 70 μl to wells in row labeled 1, 50 μl to wells in row labeled 2 and 45 μl to wells in row labeled 3. One well per row should be used per sample, whereby a single column is used per sample.
  ▴ **CRITICAL STEP** Be sure to use wells that were not already used in previous rounds if the chip is being reutilized.
- Refer to the Cell Suspension Volume Calculator table provided by the Chromium Next GEM Single Cell 5’ v2 protocol (PN-1000263) to calculate the volume of cell suspension from Step 22 and nuclease-free water to add to the master mix in Step 23. Add the calculated volume of nuclease-free water, and calculated volume of cell suspension (in that order) to the master mix, which will bring the total volume to 75 μl for each sample.
- Add 70 μl of master mix + cell suspension into the bottom center of each well in row labeled 1 of the Chromium Next GEM Chip K without introducing bubbles.
- Add 50 μl of freshly vortexed and spun-down gel beads into the wells in row labeled 2 from the Next GEM Chip K without introducing bubbles. It is very important to incubate for 30 s before moving to the next step.
- Add 45 μl of partitioning oil (provided by the Chromium Next GEM Chip K Single Cell kit, cat. no. PN-1000287) into the wells in row labeled 3 from the Next GEM Chip K.
- Attach the 10x gasket to the Next GEM Chip K, smooth the surface down and ensure the gasket holes are aligned with the wells. Avoid touching the smooth surface.
- Place the Next GEM Chip K with the gasket in the tray from the Chromium Single Cell Controller.
  ▴ **CRITICAL STEP** To avoid wobbling in the chip holder, maintain the 10x gasket assembly in a horizontal position. This could also prevent wetting of the 10x gasket with partitioning oil. Do not touch the bottom of the well in the chip with the pipette tip, load gently and pipette slowly into wells. Take care to remove any bubbles created before running the chip.
  ◆ TROUBLESHOOTING
- Run the Chip K program on the screen of the controller and press the play button.
  ▴ **CRITICAL STEP** If there are errors during single-cell acquisition, eject the tray from the controller and readjust the 10x chip holder. Ensure that the 10x gasket is properly install by aligning the holes with the wells from the Chip K.
  ◆ TROUBLESHOOTING
- After running the program (~18 min), eject the chip from the controller, discard the gasket, open the chip holder and fold the lid back at 45°.
  ▴ **CRITICAL STEP** Examine the volume in rows labeled 1 and 2 from the Chip K. Abnormally high volumes in any well indicates a potential clog.
- Slowly aspirate 100 μl of the GEMs in the wells from the row labeled 3 from the Chip K.
- Transfer the GEMs into an eight-well PCR tube strip on ice with the pipette tips against the sidewalls of the tubes. This should be done very slowly over the course of ~20 s.
  ▴ **CRITICAL STEP** The GEM solution should look opaque and uniform across all wells. Clear solutions suggest an excess of partitioning oil indicating a potential clog.
- For RT, place the eight-well PCR tube strip into the Bio-Rad C1000 Touch thermocycler and use the following settings:

**Table:** 

Lid temperature	Reaction volume		Run time
53 °C	125 μL		~55 min
		
Cycle number	RT	Denature	Final
1	53 °C, 45 min		
1		85 °C, 5 min	
Hold			4 °C
  ∎ **PAUSE POINT** Store the eight-well PCR tube strip at 4 °C for up to 72 h or at −20 °C for up to a week.
- Dispense 125 μl of the recovery agent (provided by the Chromium Next GEM Chip K Single Cell kit, cat. no. PN-1000287) into each tube at room temperature. Avoid pipetting or vortexing. Incubate for 2 min until a biphasic solution in generated in which the pink phase contains the recovery agent plus partitioning oil and the clear phase contains the aqueous solution.
  ▴ **CRITICAL STEP** If the biphasic separation is incomplete, mix the solutions by inverting the strip five times and pulse centrifuge in a tabletop mini-PCR centrifuge. Centrifugal forces can help in separating organic components based on their densities and particles sizes.
- Carefully remove and discard by pipetting 125 μl of the pink solution (recovery agent + partitioning oil) from the bottom of the tubes. Do not aspirate the aqueous solution.
  ▴ **CRITICAL STEP** The aqueous solution contains the cDNA material, and its purity depends on the removal of the pink solution that contains the partitioning oil, proteins and lipids from the cell lysis that occurred during the generation of the GEMs in Step 32.
  ◆ TROUBLESHOOTING
- Equilibrate Dynabeads at room temperature and prepare the Dynabeads Cleanup Mix as follows:

**Table:** 

Component	Amount (μl)	Final concentration
Nuclease-free water	5	1×
Cleanup buffer PN-2000088^a^	182	1×
Dynabeads MyOne SILANE PN-2000048^a^	8	1×
Reducing agent B PN-2000087^a^	5	1×
Total	200	

a Provided by the Chromium Next GEM Single Cell 5’ Reagent kits v2 (10x Genomics, cat. no. PN-1000263)
- Dispense 200 μl of the Dynabeads Cleanup Mix solution into each tube containing the aqueous solution and mix well by pipetting (set the pipette to 200 μl).
- Incubate with the Dynabeads for 10 min. Keep the caps open.
- Prepare elution solution 1 as follows:

**Table:** 

Component	Amount (μl)	Final concentration
Buffer EB	98	1×
10% Tween 20	1	0.1%
Reducing agent B	1	1×
Total	100
- Incubate the Dynabeads for 10 min at room temperature.
- Place the strip on a 10x magnetic separator in the high side (magnet•High) until the solution clears.
- Remove the supernatant by pipetting and discard.
- Dispense 300 μl of 80% ethanol onto the white pellets in each tube located in the magnet. Incubate for 30 s.
- Remove ethanol by pipetting and add 200 μl of fresh 80% ethanol to the pellets and incubate for 30 s.
- Remove ethanol by pipetting and centrifuge briefly. Place the strip on the 10x magnetic separator in low side (magnet•Low).
- Remove the excess of ethanol by pipetting and air dry the tubes for 2 min.
- Remove the strip from the magnet and immediately add 35.5 μl of the elution solution I from Step 42. Mix by pipetting (set the pipette to 30 μl) without introducing bubbles. If beads are clumpy, continue pipetting until the beads are fully resuspended. Incubate for 1 min at room temperature.
- Place the strip on the 10x magnetic separator in the low side (magnet•Low) until the solution clears. Transfer 35 μl sample to a new tube strip.
- Prepare cDNA amplification mix on ice as follows:

**Table:** 

Component	Amount (μl)	Final concentration
Amplification master mix PN-2000103^a^	50	1×
cDNA primer mix PN-2000089*	15	1×
Total	65	

a Provided by the Chromium Next GEM Single Cell 5’ Reagent kits v2 (10x Genomics, cat. no. PN-1000263)
  ▴ **CRITICAL STEP** Note that the Next GEM Single Cell 5’v2 kit has incorporated the cDNA Additive reagent into the cDNA primers and the T-cell receptor/B-cell receptor amplification primers. The cDNA additive is used to improve the efficiency of capturing diverse transcripts (length, GC content). The cDNA additive also helps to minimize the amount of formed nonspecific PCR product during enrichment steps. If using the Next GEM Single Cell 5’v2 kit PN-1000002, please follow the manufacturer’s guidelines for cDNA amplification.
- Dispense 65 μl of the cDNA amplification mix to 35 μl of sample. Mix by pipetting (set pipette to 90 μl), centrifuge briefly and incubate in the thermocycler with the following settings:

**Table:** 

Lid temperature	Reaction volume	Run time
105 °C	100 μl	~25–50 min

**Table:** 

Cycle number	Denature	Anneal	Extend	Final
1	98 °C, 45 s			
16	98 °C, 20 s	63 °C, 30 s	72 °C, 1 min	
1			72 °C, 1 min	
Hold				4 °C
  ▴ **CRITICAL STEP** If the cDNA yields are low after tumor processing and single-cell acquisition, it is advisable to increase one or two PCR cycles during the amplification steps. However, this could increase the generation of artifact products from the PCR amplification cycles. Follow the recommended number of cycles for targeted cell recovery and sample type.
  ∎ **PAUSE POINT** Store the strip at 4 °C for up to 72 h or −20 °C for ≤1 week.
  ◆ TROUBLESHOOTING

### cDNA cleanup and quantification

#### ● TIMING ~2 h

- Resuspend the SPRIselect reagent by vortexing and dispense 60 μl to each sample and mix by pipetting (set pipette to 140 μl). Incubate for 5 min at room temperature.
- Repeat Steps 44–49.
- Remove the tube strip from the magnet and dispense 45.5 μl of Buffer EB. Mix the solution by gentle pipetting and incubate for 2 min at room temperature.
- Place the tube strip on the magnet•High side until the solution clears and transfer 45 μl sample into a new tube strip.
  ∎ **PAUSE POINT** Store the tube strip at 4 °C for up to 72 h or at −20 °C for up to 4 weeks.
- Take 1 μl aliquot from each sample to quantify cDNA quality and concentration using the Agilent Bioanalyzer High Sensitivity chip.
  ▴ **CRITICAL STEP** Run 1 μl undiluted product for input cells with low RNA content (<1 pg total RNA per cell). For high RNA content, dilute the sample 1:10 with nuclease-free water and take 1 μl aliquot to measure cDNA quality and concentration.
- Calculate the volume necessary for 50 ng of cDNA for 5’ GEX library construction. If the volume required for 50 ng is less than 20 μl, adjust the total volume of each sample to 20 μl with nuclease-free water, if the volume for 50 ng exceeds 20 μl, transfer only 20 μl for library construction. Do not exceed a mass of 50 ng in the 20 μl solution.

### Generation of GEX cDNA libraries and sequencing

#### ● TIMING ~5 h

- Transfer 20 μl containing 50 ng of cDNA into a tube strip on ice. Program the thermocycler with the following settings:

**Table:** 

Lid temperature	Reaction volume	Run time
65 °C	50 μl	~35 min

**Table:** 

Cycle number	Precool block	Fragmentation	End repair and A-tailing	Final
1	4 °C, hold			
1		32 °C, 5 min		
1			65 °C, 30 min	
Hold				4 °C
- Prepare the fragmentation buffer on ice as follows:

**Table:** 

Component	Amount (μl)	Final concentration
Nuclease-free water	15	
Fragmentation buffer PN-2000091^a^	5	1×
Fragmentation enzyme PN-2000090^a^	10	1×
Total	30	

a Provided by the Chromium Next GEM Single Cell 5’ Reagent kits v2 (10x Genomics, cat. no. PN-1000263)
- After vortexing the fragmentation buffer, transfer 30 μl of the mix into the tubes containing 20 μl of cDNA from Step 59. Mix the solutions by pipetting. Centrifuge briefly and place the tube strip into the precooled (4 °C) thermocycler. Press ‘SKIP’ to run the program in Step 60.
- Vortex the SPRIselect reagent before using it. Add 30 μl SPRIselect reagent to each tube and mix by pipetting and incubate for 5 min.
- Place the tube strip into the magnet•High side until the solution clears.
- Transfer 75 μl of the supernatant into a new tube strip and add 10 μl of the SPRIselect reagent. Mix by gentle pipetting and incubate 5 min at room temperature.
- Repeat Steps 44–49.
- Repeat Steps 56–57 using 50.5 μl Buffer EB and transferring 50 μl to a new tube.
- Prepare the adaptor ligation mix as follows:

**Table:** 

Component	Amount (μl)	Final concentration
Ligation buffer PN-2000092^a^	20	1×
DNA ligase PN-220110^a^	10	1×
Adaptor oligos PN-2000094^a^	20	1×
Total	50	

a Provided by the Chromium Next GEM Single Cell 5’ Reagent Kits v2 (10x Genomics, cat. no. PN-1000263)
- Transfer 50 μl of the adaptor ligation mix to 50 μl sample. Mix by gentle pipetting (set the pipette to 90 μl) and centrifuge briefly.
- Place the tube strip into the thermocycler with the following settings:

**Table:** 

Lid temperature	Reaction volume	Run time
30 °C	100 μl	~15 min

**Table:** 

Cycle number	Condition
1	20 °C, 15 min
Hold	4 °C
- Vortex the SPRIselect reagent before using it. Dispense 80 μl SPRIselect reagent to each tube. Mix by pipetting and incubate for 5 min at room temperature.
- Repeat Steps 44–49.
- Repeat Steps 56–57 using 30.5 μl of Buffer EB, placing the tube strip on the magnet•Low side and transferring 30 μl of sample into a new tube strip.
- Select an appropriate sample index set for your samples to ensure that no sample indices overlap between samples (i.e., need a unique sample index per sample) if subsequently pooling samples for a multiplexed sequencing run. Record the 10x sample index name (PN-3000431 Dual Index Plate TT Set A well ID) used.
- Prepare sample index PCR mix as follows:

**Table:** 

Component	Amount (μl)	Final concentration
Amplification master mix PN-2000103^a^	50	1×
Dual index TT Set A PN-1000215*	20	1×
Total	70	

a Provided by the Dual Index Kit TT Set A kit (10x Genomics, cat. no. PN-1000215)
- Dispense 70 μl of the sample index PCR mix to 30 μl of sample from Step 73. Mix by pipetting (set the pipette to 90 μl) and centrifuge briefly.
- Place the tube strip into a thermocycler with the following settings:

**Table:** 

Lid temperature	Reaction volume	Run time
105 °C	100 μl	~30 min

**Table:** 

Cycle number	Denature	Anneal	Extend	Final
1	98 °C, 45 s			
16	98 °C, 20 s	54 °C, 30 s	72 °C, 20 min	
1			72 °C, 1 min	
Hold				4 °C
  ▴ **CRITICAL STEP** For samples with low cDNA content (1–25 ng) we recommend using a total of 16 cycles. For samples with higher cDNA content (16–50 ng), the total number of cycles is set to 14.
  ∎ **PAUSE POINT** Store at 4 °C for up to 72 h.
- Double-sided size selection–SPRIselect. Vortex the SPRIselect reagent before using it. Transfer 60 μl of SPRIselect reagent (0.6×) to each tube. Mix by pipetting and incubate for 5 min at room temperature.
- Place the tube strip in the magnet•High side until the solution clears and transfer 150 μl to a new tube strip.
- Dispense 20 μl of the SPRIselect reagent (0.8×) to each tube in step 79. Mix gently by pipetting and incubate for 5 min at room temperature.
  ▴ **CRITICAL STEP** The first SPRIselect (0.6×) cycle removes large cDNA fragments above 1,000 bp and the second SPRIselect (0.8×) cycle removes cDNA fragments below 200 bp, thus generating cDNA libraries between 300 and 500 bp. If a substantial proportion of the library contains <200 bp fragments, it is recommended that an additional SPRIselect cleanup be performed at 0.8× or 1× ratio.
- Repeat Steps 44–49.
- Repeat Steps 56–57 using 35.5 μl of Buffer EB, placing the tube strip on the magnet•Low side and transferring 30 μl into new a new tube strip.
  ∎ **PAUSE POINT** Store at 4 °C for up to 72 h or at −20 °C for long-term storage.
- Run 1 μl aliquot at 1:10 dilution on an Agilent Bioanalyzer High Sensitivity chip to measure cDNA quality.
- Sequence GEX libraries using an Illumina NextSeq 2000 sequencer or NovaSeq 6000 system depending on the user’s preference for sequencing reads, the number of samples being sequenced and the estimated number of cells per sample. For example, for 4,000 cells captured per sample, requiring 20,000 reads per cell we would need to obtain 80 million reads per 5’ GEX sample.

### Generation of INVADEseq bacterial 16S rRNA gene enrichment libraries

#### • TIMING ~4 h

- Prepare target enrichment 1 reaction mix on ice as follows. Vortex and centrifuge briefly.

**Table:** 

Component	Amount (μl)	Final concentration
Nuclease-free water	8	
Amplification master mix PN-220125^a^	50	1×
cDNA additive PN-220067^a^	5	1×
16S_Enrich_Forward (100 μM)	1	1.5 μM
1061R Enrich_Nested_Reverse (100 μM)	1	1.5 μM
Total	65	

a Provided by the Chromium Single Cell 5’ Library kit, PN-1000002
  ▴ **CRITICAL STEP** 10x Genomics 5′ v2 scRNAseq kit has incorporated the cDNA Additive reagent into the cDNA primers and the T-cell receptor/B-cell receptor amplification primers. The cDNA additive is used to improve the efficiency of capturing diverse transcripts (length, GC content). The cDNA additive also helps to minimize the amount of nonspecific PCR product formed during enrichment steps. If using the 10x Genomics 5’ v2 kit PN-1000002, an alternative to the cDNA Additive reagent is Lucigen’s MasterAmp 10x PCR Enhance (product number: ME81210), added at the same volume as recommended for the cDNA Additive.
- Dilute between 2 and 10 μl of amplified cDNA generated from the 10x Genomics Chromium Single Cell kit CG000086-RevJ from Step 82 to a final volume of 35 μl with nuclease-free H_2_O in a PCR tube and then add 65 μl target enrichment 1 reaction mix from Step 85 to each PCR tube containing the diluted cDNA. The final volume is 100 μl.
  ▴ **CRITICAL STEP** We note a range of 2–10 μl of cDNAas an input to allow to user flexibility depending on their specimen type and estimation of bacterial load. For samples with expected high microbial load, we would suggest an input of 2 μl of cDNA (to be diluted to a final volume of 35 μl) but for samples with very low microbial load an input of 10 μl of cDNA (to be diluted to a final volume of 35 μl) may be required. Most of the input cDNA is mammalian host cDNA and this step is to enrich the minor bacterial component within this cDNA mix.
- Mix by pipetting (set the pipette to 80 μl) and centrifuge briefly.
- Place the tubes in a thermocycler using the following settings:

**Table:** 

Cycle number	Denature	Anneal	Extend	Final
1	98 °C, 45 s			
35	98 °C, 20 s	67 °C (ramp rate 2 °C/s), 30 s	72 °C, 1 min	
1			72 °C, 1 min	
Hold				4 °C
  ∎ **PAUSE POINT** Store at 4 °C for up to 72 h or at −20 °C for long-term storage.
- Repeat Step 71.
  ▴ **CRITICAL STEP** Single-sided SPRIselect is performed in Step 89 instead of the standard double-sided SPRIselect performed in Steps 78–80 to retain the larger cDNA fragments that include the bacterial transcripts.
- Repeat Steps 44–49.
- Repeat Steps 56–57 using 37 μl of Buffer EB, placing the strip on the magnet•Low side and transferring 35 μl to a new tube strip.
- Add 10 μl of BluePippin internal standard mix to each tube from Step 91.
- Prepare the 1.5% agarose gel cassette for selecting the enriched product by size using the BluePippin platform (Sage Sciences).
  ▴ **CRITICAL STEP** Place the cassette horizontally and make sure the reservoirs are nearly full of electrophoresis buffer or the buffer level are the same across the cassette. Low reservoir levels should be refilled with the supplied electrophoresis buffer before running. Verify the presence of bubbles by turning the cassette upside down.
- Place the cassette into the BluePippin optical nest.
- Remove buffer from the elution modules and replace with 40 μl of fresh electrophoresis buffer.
- Seal the elution wells with the adhesive tape strips.
- Verify that the sample wells are filled with 70 μl of electrophoresis buffer.
- Close the lid and press the ‘TEST’ button. A continuity test is automatically run to measure the current in each separation lane.
  ▴ **CRITICAL STEP** Do not use a lane if a separation lane has failed (‘FAIL’ message highlighted in orange) the continuity test. Remaining passing lanes can be used if necessary.
- Open the lid and remove 40 μl of buffer from one of the sample wells and add 40 μl of the sample into the well while the cassette is placed into the nest.
- Select the ‘Tight’ programming mode to select products between 955 and 1,215 bp, assign the ‘USE INTERNAL STANDARDS’ option and run the program.
  ▴ **CRITICAL STEP** Inaccurate size selection can result in loss of the bacterial enrichment library.
  ▴ **CRITICAL STEP** Broaden the collection range of the distribution size (bp) of cDNA fragments before running the ‘Tight’ program to increase the overall DNA content.
  ◆ TROUBLESHOOTING
- Remove the sealing tape strip that covers the elution well.
- Collect 35 μl of sample in electrophoresis buffer from the elution well.
- Prepare target enrichment 2 reaction mix on ice as follows. Vortex and centrifuge briefly.

**Table:** 

Component	Amount (μl)	Final concentration
Nuclease-free water	8	
Amplification master mix PN-220125^a^	50	1×
cDNA Additive PN-220067^a^	5	1×
16S_Enrich_Forward primer (100 μM)	1	1.5 μM
1061R Enrich_Nested_Reverse (100 μM)	1	1.5 μM
Total	65	

a Provided by the Chromium Single Cell 5’ Library kit, 16 reactions PN-1000002.
- Add 65 μl target enrichment 2 reaction mix to each tube containing 35 μl sample from the selection process in Step 102.
- Mix by pipetting (set the pipette to 80 μl) and centrifuge briefly.
- Place the tubes in a thermocycler using the following settings:

**Table:** 

Cycle number	Denature	Anneal	Extend	Final
1	98 °C, 45 s			
20	98 °C, 20 s	67 °C (ramp rate 2 °C/s), 30 s	72 °C, 1 min	
1			72 °C, 1 min	
Hold				4 °C
  ∎ **PAUSE POINT** Store at 4 °C for up to 72 h or at −20 °C for long-term storage.
- Repeat Step 71.
- Repeat Steps44–49.
- Repeat Steps56–57 using 37 μl of Buffer EB, placing the strip on the magnet·Low side and transferring 35 μl to a new tube strip.
- Run 1 μl sample at 1:5 dilution (dilution factor 5) with nuclease-free water on an Agilent Tapestation 4200 using the Agilent D5000 ScreenTape to measure enrichment product concentrations (Agilent Technologies).
- Determine the corresponding volume for a total of 50 ng of the second enrichment product. If the volume required for 50 ng is less than 30 μl, adjust the total volume of each sample to 30 μl with nuclease-free water. If the volume for 50 ng exceeds 30 μl, carry only 30 μl sample for PCR indexing.
  ▴ **CRITICAL STEP** If the cDNA yields are low after the washing steps, it is recommended to increase the amplification step by one or two PCR cycles.
  ♦ TROUBLESHOOTING
- Choose the appropriate sample index sets per the 10x Genomics Single Cell protocol CG000086-RevJ.
- Prepare sample index PCR mix as follows:

**Table:** 

Component	Amount (μl)	Final concentration
Amplification master mix PN-2000103^a^	50	1×
Dual index TT Set A PN-1000215^a^	20	1×
Total	70	

a Provided by the Dual Index Kit TT Set A, 96 reactions (10x Genomics, cat. no. PN-1000215).
- Add 70 μl of sample index PCR mix to 30 μl sample (50 ng) from Step 111, for a final volume of 100 μl.
- Place the tube samples into a thermocycler with the following settings:

**Table:** 

Lid temperature	Reaction volume	Run time
105 °C	100 μl	~30 min

**Table:** 

Cycle number	Denature	Anneal	Extend	Final
1	98 °C, 45 s			
15	98 °C, 20 s	54 °C, 30 s	72 °C, 20 min	
1			72 °C, 1 min	
Hold				4 °C
  ∎ **PAUSE POINT** Store at 4 °C for up to 72 h or proceed to the next step.
- Repeat Step 71.
- Repeat Steps44–49.
- Repat Steps 56–57 using 37 μl of Buffer EB, placing the strip on the magnet•Low side and transferring 35 μl to a new tube strip.
  ∎ **PAUSE POINT** Store at 4 °C for up to 72 h or at −20 °C for long-term storage.
- Repeat Steps 92–102 adding 10 μl of BluePippin internal standard mix to 35 μl of sample from Step 118.
- Quantify library size distributions by using the Agilent High Sensitivity D5000 ScreenTape (for an example of library size distribution, see Supplementary Fig. 1). Additional library quality control (QC), blending of pooled indexed libraries and cluster optimization is performed using the KAPA Quantification kit for Illumina (Roche Sequencing and Life Science) following the manufacturer’s instructions.
- Sequence libraries on a MiSeq (lllumina) employing a paired-end, 300 base read length (PE300), using V3 reagents and multiplexing between seven and nine samples per flow cell.
  ▴ **CRITICAL STEP** increasing the number of sequencing reads per single cell increases the probability of capturing transcripts of low abundance such as bacterial transcripts. The INVADEseq bacterial 16S rRNA gene libraries facilitate the enrichment of bacterial transcripts but these bacterial transcripts will still be a minor component of the sequencing library compared to the total mammalian transcripts (Fig. 2).
  ♦ TROUBLESHOOTING
- Perform on the instrument, a secondary analysis with MiSeq Reporter Software v2.5.1 (Illumina) using basecalls and quality scores generated by RTA v1.18.54 (Illumina). The MiSeq Reporter Software v2.5.1 is preinstalled on MiSeq sequencers and its function is to process base calls generated on-instrument during the sequencing run by RTA software. When the RTA is completed, the MiSeq Reporter will launch automatically. Please refer to the Illumina MiSeq Reporter Software Reference Guide for user-defined secondary analysis, not related to INVADEseq, such as alignments to reference genomes and contig generation.

### Computational analysis of single-cell sequencing data Acquiring databases

#### ● TIMING ~1 h

- Download the human reference genome (GRCh38) and mouse reference genome for CellRanger analysis from 10x Genomics (https://support.10xgenomics.com/single-cell-gene-expression/software/downloads/latest).
- Download prebuilt host and microbe reference files for PathSeq analysis from the Broad FTP server (https://software.broadinstitute.org/pathseq/Downloads.html).

### Generation of bacteria UMI matrixes from the GEX library

#### ● TIMING ~3h

- Copy or link the sample folder containing raw 10x FASTQ files that generated by cellranger mkfastq to the raw data folder. In this protocol, the example input folder is:
  $/invadeseq/raw_data/GEX/
- Change the working directory to a folder you would like to save CellRanger results in. In this protocol, the example CellRanger output path is:
  $/invadeseq/GEX_data_process/cellranger_count/
- Process raw FASTQ files and map reads to the reference genome using CellRanger:
  $ cellranger count --id=sample_name --transcriptome=CellrangerDatabaseDir --fastqs=/ invadeseq/raw_data/GEX/SampleX --sample=SampleX
- Process the CellRanger generated BAM file using GATK PathSeq as follows:
  $ gatk PathSeqPipelineSpark --input /invadeseq/GEX_data_process/cellranger_count/ SampleX/outs/possorted_genome_bam.bam --filter-bwa-image PathSeqDB/pathseq_ host.fa.img --kmer-file PathSeqDB/pathseq_host.bfi --min-clipped-read-length 60 --microbe-fasta PathSeqDB/pathseq_microbe.fa --microbe-bwa-image PathSeqDB/ pathseq_microbe.fa.img --taxonomy-file PathSeqDB/pathseq_taxonomy.db --output / invadeseq/GEX_data_process/pathseq_results/SampleX.pathseq.bam --scores-output / invadeseq/GEX_data_process/pathseq_results/SampleX.pathseq.csv --is-host-aligned false --filter-duplicates false --min-score-identity.7
  ▴ **CRITICAL STEP** The amount of memory to run PathSeq can be specified (for example, 750Gb) by adding --java-options “-Xmx750g” before PathSeqPipelineSpark: $ gatk --java-options “-Xmx750g” PathSeqPipelineSpark.
- Within the PathSeq data output from the GEX libraries, locate the file path for the PathSeq generated files that include a BAM file named SampleX.pathseq.bam that contains the bacterial annotation and a summary CSV file named SampleX.pathseq.csv.
- Then run the INVADESeq Python script using the BAM files generated in the previous steps: $ python INVADESeq.py \
  /invadeseq/GEX_data_process/cellranger_count/SampleX/outs/possorted_genome_bam.bam \ SampleX \
  /invadeseq/GEX_data_process/cellranger_count/SampleX/outs/filtered_feature_bc_ matrix/barcodes.tsv.gz \
  /invadeseq/GEX_data_process/pathseq_results/SampleX.pathseq.bam \
  /invadeseq/GEX_data_process/pathseq_results/SampleX.pathseq.csv \
  /invadeseq/GEX_data_process/invadeseq_results/SampleX.gex.readname \
  /invadeseq/GEX_data_process/invadeseq_results/SampleX.gex.unmap_cbub.bam \
  /invadeseq/GEX_data_process/invadeseq_results/SampleX.gex.unmap_cbub.fasta \
  /invadeseq/GEX_data_process/invadeseq_results/SampleX.gex.list \
  /invadeseq/GEX_data_process/invadeseq_results/SampleX.gex.raw.readnamepath \
  /invadeseq/GEX_data_process/invadeseq_results/SampleX.gex.genus.cell \
  /invadeseq/GEX_data_process/invadeseq_results/SampleX.gex.genus.csv \
  /invadeseq/GEX_data_process/invadeseq_results/SampleX.gex.validate.csv
  ▴ **CRITICAL STEP** The file gex.genus.csv contains the GEX library and bacteria UMI matrixes, the SampleX.gex.validate.csv file contains the UMI levels and bacteria cell annotations. The SampleX.gex.raw.readnamepath file contains read-level annotation.

### Generation of bacteria UMI matrixes from the bacterial 16S rRNA gene enrichment library

#### ● TIMING ~3 h

- Copy or link the sample folder containing raw FASTQ files generated by cellranger mkfastq to the raw data folder /invadeseq/raw_data/16s/ as an example.
- Change the directory to a folder you would like to save CellRanger results. Assume the CellRanger output path is: $ /invadeseq/16s_data_process/cellranger_count/
- Process raw FASTQ files and map reads to the reference genome using CellRanger:
  $ cellranger count --id=SampleX --transcriptome=CellrangerDatabaseDir --fastqs=/ invadeseq/raw_data/16s/SampleX -sample=SampleX
  ▴ **CRITICAL STEP** Apply --chemistry=SC5P-PE if you are using CellRanger.
- Change directory to the folder you would like to save FASTQ files that will be converted from BAM files:
  $ cd /invadeseq/16s_data_process/split_reads/
- Convert the BAM file to FASTQ format :
  $ bedtools bamtofastq -i /invadeseq/16s_data_process/cellranger_count/SampleX/outs/ possorted_genome_bam.bam -fq SampleX.r1.fq -fq2 SampleX.r2.fq
- Run Trimmomatic on Read 1 file to remove adapter sequences and low-quality bases:
  $ java -jar trimmomatic-0.39.jar SE \
  SampleX.r1.fq \
  SampleX.SE_trim.fq \
  ILLUMINACLIP:$EBROOTTRIMMOMATIC/adapters/TruSeq3-PE-2.fa:2:30:10 \
  LEADING:3 TRAILING:3 SLIDINGWINDOW:4:15 MINLEN:36 HEADCROP:15
  ▴ **CRITICAL STEP** The number of threads can be adjusted by -threads. Adapter file TruSeq3-PE-2.fa is available at https://github.com/timflutre/trimmomatic/blob/master/adapters/TruSeq3-PE-2.fa.
- Convert trimmed Read 1 file from FASTQ format to BAM format using Picard FastqToSam:
  $ java -jar picard.jar FastqToSam \
  FASTQ= SampleX.SE_trim.fq \
  OUTPUT= SampleX.r1.bam \
  READ_GROUP_NAME= SampleX \
  SAMPLEX= SampleX
- Move generated BAM file:
  $ mv SampleX.r1.bam /invadeseq/16s_data_process/ubams_r1
- Process Read 1 BAM file using GATK PathSeq:
  $ gatk PathSeqPipelineSpark --input /invadeseq/16s_data_process/ubams_r1/SampleX. r1.bam --filter-bwa-image PathSeqDB/pathseq_host.fa.img --kmer-file PathSeqDB/ pathseq_host.bfi --min-clipped-read-length 60 --microbe-fasta PathSeqDB/pathseq_ microbe.fa --microbe-bwa-image PathSeqDB/pathseq_microbe.fa.img --taxonomy-file PathSeqDB/pathseq_taxonomy.db --output /invadeseq/16s_data_process/pathseq_ results/SampleX.pathseq.bam --scores-output /invadeseq/16s_data_process/pathseq_ results/SampleX.pathseq.csv --is-host-aligned false --filter-duplicates false --min-score-identity.7
- Within the PathSeq data output from the bacterial 16S rRNA gene enrichment library, locate the file path for the PathSeq generated files that include a BAM file named SampleX. pathseq.bam that contains the bacterial annotation and a summary CSV file named SampleX.pathseq.csv.
- Run the INVADESeq Python script with CellRanger BAM file and PathSeq BAM file from the bacterial 16S rRNA gene enrichment library generated in the previous steps:
  $ python INVADESeq.py \
  /invadeseq/16s_data_process/cellranger_count/SampleX/outs/possorted_genome_bam. bam \
  SampleX \
  /invadeseq/GEX_data_process/cellranger_count/SampleX/outs/filtered_feature_bc_matrix/barcodes.tsv.gz \
  /invadeseq/16s_data_process/pathseq_results/SampleX.pathseq.bam \
  /invadeseq/16s_data_process/pathseq_results/SampleX.pathseq.csv \
  /invadeseq/16s_data_process/invadeseq_results/SampleX.16s.readname \
  /invadeseq/16s_data_process/invadeseq_results/SampleX.16s.unmap_cbub.bam \
  /invadeseq/16s_data_process/invadeseq_results/SampleX.16s.unmap_cbub.fasta \
  /invadeseq/16s_data_process/invadeseq_results/SampleX.16s.list \
  /invadeseq/16s_data_process/invadeseq_results/SampleX.16s.raw.readnamepath \
  /invadeseq/16s_data_process/invadeseq_results/SampleX.16s.genus.cell \
  /invadeseq/16s_data_process/invadeseq_results/SampleX.16s.genus.csv \
  /invadeseq/16s_data_process/invadeseq_results/SampleX.16s.validate.csv
  ▴ **CRITICAL STEP** We use barcodes.tsv.gz from GEX library to process actual cells from GEX library instead of 16S bacterial enrichment library.

### Merge bacteria UMI matrices from GEX and bacterial 16S rRNA gene enrichment library

#### ● TIMING ~15 min

- Copy UMI matrices (SampleX.gex.genus.csv and SampleX.16s.genus.csv) and validation files (SampleX.gex.validate.csv and SampleX.16s.validate.csv) into the same folder:
  $ cp /invadeseq/gex_data_process/invadeseq_results/SampleX.gex.genus.csv /invadeseq/ merge_matrices/
  $ cp /invadeseq/16s_data_process/invadeseq_results/SampleX.16s.genus.csv /invadeseq/ merge_matrices/
- Run the Python script for merging matrices:
  $ python merge_metadata.py /invadeseq/merge_matrices
- Run the Python script for UMI deduplication in merged bacteria UMI matrix:
  $ python metadata_dedup.py metadata_dedup.py
- /invadeseq/merge_matrices/csv_novami.csv is the merged bacteria UMI matrix that can be loaded for downstream single-cell analysis.
  ▴ **CRITICAL STEP** Multiple samples can be merged at once.
  ▴ **CRITICAL STEP** To maximize the successful application of the INVADEseq approach to a particular specimen or disease type with unknown microbial load, confirmation of intracellular or cell-associated bacteria via RNAscope imaging with a eubacterial probe is recommended.
  ♦ TROUBLESHOOTING

### Single-cell analysis and mapping of bacteria-associated cells using Seurat

#### ● TIMING ~20 min

- Start R environment: $ R version 4.2.0 (2022-04-22) – ‘Vigorous Calisthenics’
  Copyright (C) 2022 The R Foundation for Statistical Computing
  Platform: x86_64-pc-linux-gnu (64-bit)
- Load R packages:
  > library(harmony)
  > library(Seurat)
  > library(SingleR)
  > library(celldex)
  > library(msigdbr)
  > library(clusterProfiler)
- Load single-cell data into Seurat:
  > SampleX.data<-Read10X(data.dir = “ /invadeseq/GEX_data_process/cellranger_count/SampleX/outs/filtered_feature_bc_matrix/”)
  > SampleX = CreateSeuratObject(counts = SampleX.data, project = “SampleX”, min.cells = 3, min.features = 200)
- Calculate mitochondria gene content for optional QC:
  > SampleX [[“percent.mt”]] <- PercentageFeatureSet(SampleX, pattern = “^MT-”)
  ▴ **CRITICAL STEP** This protocol applies the functions min.cells = 3 and min.features = 200 as thresholds to perform QC, which maintains cells that express more than 200 detected genes and gene transcripts detected in more than 3 cells. It is optional to perform extra QC steps, for example, remove doublets and remove cells with high mitochondria gene content.
  ♦ TROUBLESHOOTING
- Merge samples and add sample names to cell barcodes:
  > Sample_Tumors <- merge(SampleX,y=(SampleY,SampleZ), add.cell.ids = c(‘SampleX’, ‘SampleY’,’SampleZ’))
  ▴ **CRITICAL STEP** If only one sample is being processed, use RenameCells to add sample name to cell barcodes:
  > SampleX<- RenameCells(object = SampleX, add.cell.id = ‘SampleX’)

Then continue to use SampleX as sample name instead of Sample_Tumors in the following steps.

- Perform data normalization using NormalizeData function:
  > Sample_Tumors <- NormalizeData(object = Sample_Tumors, normalization.method = “LogNormalize”, scale.factor = 10000)
- Find the most variable genes across cells:
  > Sample_Tumors <- FindVariableFeatures(object = Sample_Tumors, selection.method = “vst”,nfeatures = 5000)
- Scale single-cell data using all genes:
  >all.genes <- rownames(Sample_Tumors)
  > Sample_Tumors <- ScaleData(object = Sample_Tumors,features = all.genes)
- Run principal component analysis (PCA) on single-cell data:
  > Sample_Tumors <- RunPCA(object = Sample_Tumors, pc.genes = VariableFeatures(Sample_Tumors))
- Integrate different samples using Harmony:
  > Sample_Tumors <- RunHarmony(Sample_Tumors, group.by.vars=“orig.ident”, assay. use=‘RNA’)
- Run uniform manifold approximation and projection (UMAP) analysis on the integrated sample:
  > Sample_Tumors <- RunUMAP(Sample_Tumors, dims = 1:20, reduction = “harmony”)
- Cluster the cells using FindNeighbors and FindNeighbors functions:
  > Sample_Tumors <- FindNeighbors(Sample_Tumors, reduction = “harmony”, dims = 1:20)
  > Sample_Tumors <- FindClusters(Sample_Tumors, resolution = 0.5)

### Cell-type prediction using SingleR

#### ● TIMING ~1 h

- Load Human Primary Cell Atlas Data as the reference:
  > ref <- celldex::HumanPrimaryCellAtlasData()
  ▴ **CRITICAL STEP** Alternative databases can be obtained such as Blueprint and ENCODE, Database of Immune Cell Expression (DICE) and Sorted human immune cells reference dataset (Monaco Immune Data) as follows:
  > ref <- celldex::BlueprintEncodeData()
  Database of Immune Cell Expression (DICE) reference dataset:
  > ref <- celldex::DatabaseImmuneCellExpressionData() Sorted human immune cells reference dataset:
  > ref <- celldex::MonacoimmuneData()
  The selection of a reference dataset plays an important role in the cell annotation of single-cell data according to specific functional requirements at different levels of granularity.
- Perform SingleR cell-type prediction based on fine labels from reference data:
  > seuratObj_annot <- as.SingleCellExperiment(Sample_Tumors)
  > pred <- SingleR(test=seuratObj_annot, ref=ref, cluster=seuratObj_annot@ colData$seurat_clusters, labels=ref$label.fine)
  > Sample_Tumors[[“SingleR.cluster.labels”]] <-
  pred$labels[match(Sample_Tumors[[]][[“seurat_clusters”]], rownames(pred))]

### Add bacteria UMI matrix to single-cell data

#### ● TIMING ~5 min.

- Read the bacteria UMI csv table as a dataframe:
  > umi_table_csv = ‘/invadeseq/merge_matrices/csv_novami.csv’
  > umi_table <- read.csv(umi_table_csv, sep=‘,’, header=TRUE, row.names = 1)
- Calculate the sum of bacteria UMI for each cell in a UMi_table:
  > umi_table$Total <- rowSums(umi_table)
- Map bacteria annotation to integrated single-cell data:
  > Sample_Tumors <-AddMetaData(Sample_Tumors, umi_table)
  > Sample_Tumors @meta.data[is.na(Sample_Tumors @meta.data)] <- 0
  ▴ **CRITICAL STEP** The INVADEseq computational pipeline assesses Read 1 for bacterial taxonomic assignment. Validation of the taxonomic assignment with Read 2 may increase the stringency of taxa assignment. Applying in silico microbiome contamination prediction tools may help in distinguishing contaminants from true specimen-associated reads.
  ♦ TROUBLESHOOTING

### Gene expression analysis and GSEA

#### ● TIMING ~45 min.

- Group cells based on bacteria UMI count. In this protocol, we define cells with more than four Fusobacteria UMI as ‘Fusobacterium Positive’ cells, cells without bacteria UMI as ‘Bacteria Negative’ cells, and other cells as NA. This information will be stored in a new column ‘Fuso_pos’ in single-cell metadata.
  > Sample_Tumors@meta.data$Fuso_pos <- ifelse(Sample_Tumors@meta.data$Total==0, “Bacteria Negative”, ifelse(Sample_Tumors@meta.data$Fusobacterium >=4, “Fusobacterium Positive”,NA))
- Run differential analysis to compute differently expressed genes between Fusobacterium Positive cells and Bacteria Negative cells:
  > seurat_object.markers <- FindMarkers(Sample_Tumors,
  ident.1 = “Fusobacterium Positive”,
  ident.2 = “Bacteria Negative”,
  group.by = “Fuso_pos”,
  logfc.threshold = -Inf,
  min.pct = 0.1)
- Prepare Hallmark gene sets for GSEA:
  > m_H <- msigdbr(species = “Homo sapiens”, category = “H”) %>% dplyr::select(gs_name, gene_symbol)
- Prepare a ranked gene list based on average log2 fold change
  > gene_list_seurat_object <- seurat_object.markers[,c(“avg_log2FC”)]
  > names(gene_list_seurat_object) = as.character(rownames(seurat_object.markers))
  > gene_list_seurat_object = sort(gene_list_seurat_object, decreasing = TRUE)
- Perform GSEA analysis based on Hallmark gene sets:
  markers_seurat_object.em <- GSEA(markers_seurat_object, TERM2GENE = m_H, eps=0.0, by = “fgsea”)

### (Optional) Generation of bacterial UMI matrix using the Nextflow pipeline

#### ● TIMING ~5 h

▴ **CRITICAL** The generation of a bacterial UMI matrix using the Nextflow pipeline (Steps 168–176) is an alternative option that offers a simplified and automated approach to analyze single-cell sequencing data. Users can use this option if they prefer to streamline the data processing, reduce manual intervention and minimize potential errors. However, researchers can also choose to analyze the data using a hands-on approach in executing individual steps according to their requirements. To run the INVADEseq Nextflow pipeline, the user must provide single-cell sequencing data produced by the 10x Chromium platform, both for GEX and for 16S-enriched sequences contained in the FASTQ files.

- Create a manifest file to specify the location of the input data. This file should be in CSV format, listing the paired datasets produced from each sample, with columns named ‘sample’, ‘gex’ and ‘microbial’.
  For example:
  sample,gex,microbial
  SampleX,SampleX_gex,SampleX_microbial
  SampleY,SampleY_gex,SampleY_microbial
  SampleZ,SampleZ_gex,SampleZ_microbial
- Input the FASTQ files, produced using the ‘cellranger mkfastq’ command, into this workflow. Ensure these files are tagged with the sample names used during the 10x Chromium platform preparation and contained within a shared directory.
- Use the parameter ’manifest’ to provide the manifest file to the workflow.
- Use the parameter ’fastq_dir’ to supply the root folder that contains all of the FASTQ files for the analysis. Note that any files with the extension. fastq.gz can be used in the analysis, even if they are nested within additional subfolders.
- Install BASH Workbench (https://github.com/FredHutch/bash-workbench/wiki): $ pip3 install bash-workbench.
- (Optional) Set up container dependencies using Docker or Singularity/Apptainer.
  These are additional resources to manage dependencies more effectively.
  ▴ **CRITICAL STEP** For single-user systems, Docker is an alternative resource that can simplify the process of managing and loading dependencies. Docker Desktop can be installed following directions at https://www.docker.com/products/docker-desktop/. However, users might choose to skip this step if they have all necessary dependencies installed natively on their own systems or prefer to manage dependencies manually. For multi-user systems, it is recommended to use the SLURM scheduler (https://www.schedmd.com/) along with the Singularity/Apptainer applications (https://sylabs.io/ and https://apptainer.org/) to manage dependencies more effectively due to its better compatibility with shared resources. However, if the scheduler is not available or the user has administrative control over software installations, this step could be bypassed.
- To set up this workflow in the BASH Workbench, select:
  - Select Manage Repositories
  - Select Download New Repository
  - Then enter FredHutch/invadeseq and confirm
  - After setting up the workflow, the workbench can be exited with Control + C
- After setting up the workflow, run it by:
  - Navigating to the folder intended for the output flies
  - Launching the BASH Workbench (wb)
  - Select Run Tool
  - Select FredHutch invadeseq
  - Select invadeseq
  - Enter the appropriate parameters
  - Select Review and Run
  - Select FredHutch invadeseq
  - Select slurm (if using a high-performance computing SLURM cluster) or docker (for local execution)
  - Enter any needed parameters for the SLURM or Docker configuration. For example, SLURM users will need to enter the scratch dir parameter using a folder on the scratch filesystem which can be used for temporary files
  - Select Review and Run
  - Select Run Now
- Once the workflow has been launched, a record will be saved of the parameters used for execution, as well as all of the logs that were produced during execution.
  - csv_novami.csv # Combined genus-level counts per cell
  - gex/ # Combined gene expression counts per cell
  - <SAMPLE>/# Folder with sample-level results
  - pathseq_16S/ # PathSeq results from 16S data
  - pathseq_gex/ # PathSeq results from GEX data
  - cellranger 16S/ # CellRanger results from 16S data
  - cellranger_gex/ # CellRanger results from GEX data
  - preqc/ # FASTQC results for 16S (R1) data pre-trimming
  - postqc/ # FASTQC results for 16S (R1) data post-trimming
  - umi_16S/ # UMI summary metrics for 16S data
  - umi_gex/ # UMI summary metrics for GEX data

## Troubleshooting

Troubleshooting advice can be found in Table 1.

## Timing

Steps 1–22, tumor dissociation for single-cell generation: ~2 h

Steps 23–53, single-cell acquisition for RNA sequencing: ~4 h

Steps 54–59, cDNA cleanup and quantification: ~2 h

Steps 60–84, generation of GEX cDNA libraries and sequencing: ~4 h

Steps 85–122, generation of INVADEseq bacterial 16S rRNA gene libraries: ~5 h

Steps 123 and 124, acquiring databases: ~1 h

Steps 125–130, generation of bacteria UMI matrixes from the GEX library: ~3 h

Steps 131–141, generation of bacteria UMI matrixes from the 16S bacterial enrichment library: ~3 h

Steps 142–145, merge bacteria UMI matrices from GEX and 16S bacterial enrichment libraries: ~15 min

Steps 146–157, single-cell analysis and bacteria-associated cells mapping using Seurat: ~20 min

Steps 158 and 159, cell-type prediction using SingleR: ~1 h

Steps 160–162, add bacteria UMI matrix to single-cell data: ~5 min

Steps 163–167, gene expression analysis and GSEA: ~45 min

Steps 168–176, (optional) generate bacteria UMI matrix using Nextflow pipeline: ~5 h

## Anticipated results

We have previously shown that the INVADEseq approach is a reliable tool to reveal the identity of cell adherent and cell invasive bacteria, the host cell types they interact with and their impact on transcriptional programs of human single cells within tumor tissue^8^. Our application of the INVADEseq approach to human OSCC tumor tissues demonstrated that cell-associated intratumoral bacteria were largely detected within a subset of epithelial and macrophage single cells. Additionally, we observed that *Fusobacterium* and *Treponema* species were the dominant cell associated bacteria in OSCC tumors. Comparison of gene expression within the epithelial single-cell clusters or macrophage cell clusters based on bacterial presence or absence can reveal cell-type-specific genes and pathways altered by these microbial components. Comparisons can be performed at different bacterial taxonomic resolutions, ranging from the bacterial kingdom (general bacteria positive) to bacterial species level, although in our previous analysis we have limited the resolution to genus level due to genus level conservation of variable regions within the 16S rRNA gene.

It is possible that single cells from tumor samples at mucosal sites may have a high prevalence of cell-associated bacteria within specific cell types such as macrophages. In these cases, because the proportion of bacteria-associated cells are largely abundant, the statistical power to identify differentially expressed genes may be limited due to a low number of bacteria-negative single cells. In such cases, researchers can integrate INVADEseq single-cell sequencing data from additional comparative specimens to increase single-cell numbers for cell types and provide a comparative bacteria-negative cell population. Comparative gene expression analysis of single cells based on bacterial status can be performed with specific bacterial taxa or at the bacterial kingdom level, which may dilute taxa-specific patterns but reveal shared genes that are differently expressed by the dominant cell-associated taxa.

**Fig. 3 |:**
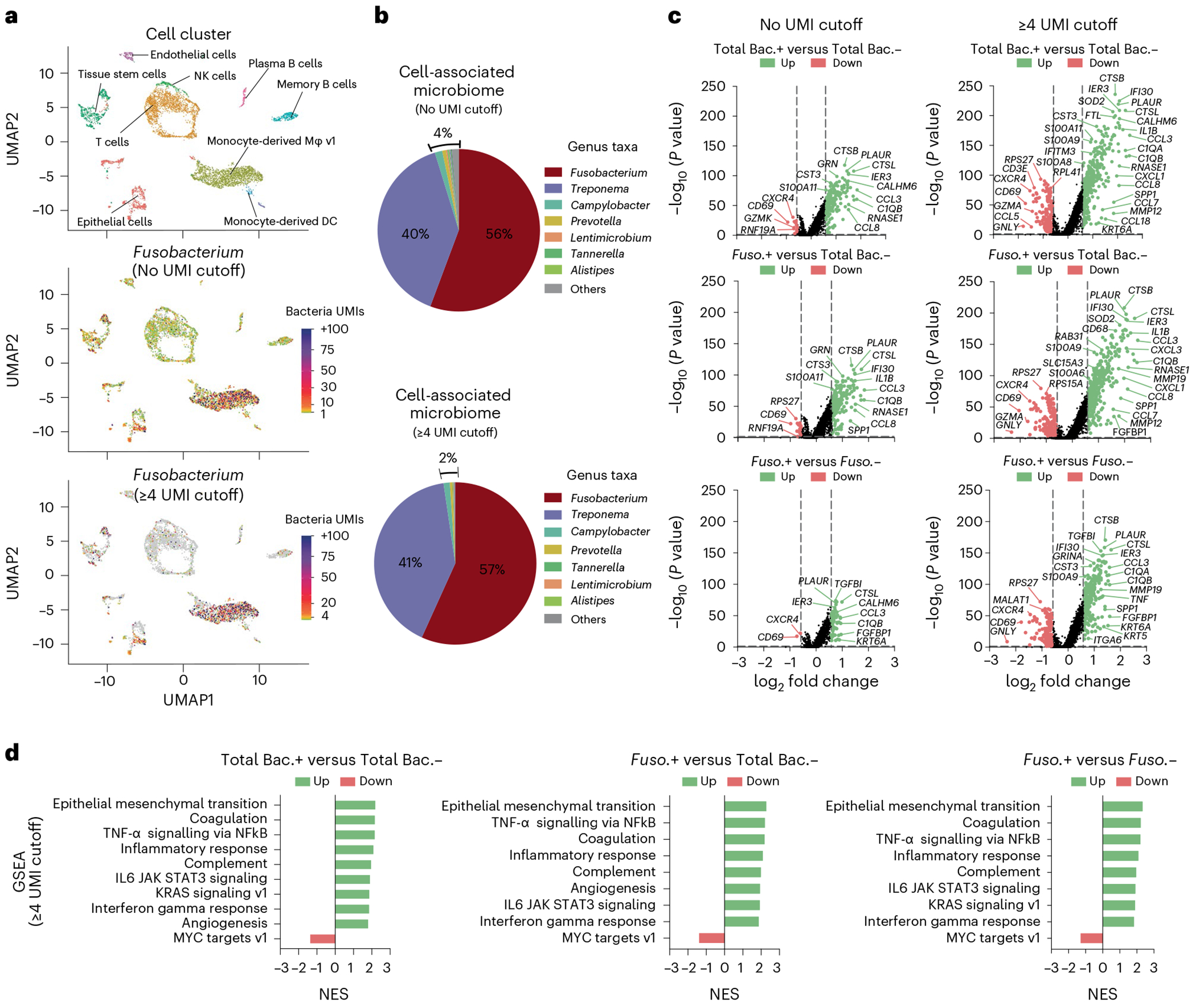
Single-cell RNAseq analysis of a OSCC tumor sample using the INVADEseq method. **a,** UMAP plots showing the eukaryotic cellar composition and the distribution of *Fusobacterium* transcripts by applying or not a ≥4 UMI threshold as indicated. Color bars indicate the transcriptional UMI bacteria load. **b**, Cell-associated microbiome showing the most frequent bacterial communities in the tumor tissue from an OSCC case by implementing or not an ≥4 UMI threshold. See also Supplementary Table 1. **c**, Volcano plots showing the differentially expressed genes by comparing the following eukaryotic cell populations and by using or not an ≥4 UMI threshold as indicated. Top: total bacteria-positive cells (Total Bac.+) against total bacteria-negative cells (Total Bac.−). Middle: *Fusobacterium*-positive cells (*Fuso*.+) against total bacteria-negative cells (Total Bac,−). Bottom: *Fusobacterium*-positive cells (*Fuso.*+) against *Fusobacterium*-negative cells (*Fuso.*−). Dashed lines indicate the threshold of significant gene expression defined as the log2 fold change ≤−0.58 and ≥0.58 with a −log_10_ *P* value ≥1.301. Fold changes and *P* values were calculated by using a linear mixed model (LMM), followed by a Benjamini–Hochberg multiple correction test. See also Supplementary Table 2. **d**, GSEA analysis showing the signaling pathways that are differentially regulated by comparing the cell populations described in **c** and by applying an UMI threshold of ≥4. A Wilcoxon rank sum test was implemented to calculate the normalized enrichment score (NES). See also Supplementary Table 3. The data used to generate this figure have been published previously^8^. NK, natural killer cells; DC, dendritic cells.

For intra-patient single-cell analysis independent of cell type, host transcriptional changes induced by cell-associated bacteria can be identified by comparing bacteria-associated single cells (Total Bac.+) to bacteria-negative single cells (Total Bac.−) in the entire single-cell sample (Fig. 3a). Additionally, by using the UMI metric as a proxy for transcriptional load, researchers can establish different thresholds to identify and compare host single-cell populations based on bacterial UMI levels. For instance, by applying a bacterial UMI threshold of ≥4, a host cell population or type that harbors relatively high levels of bacterial transcripts are identified and can be compared with single-cell populations that contain lower levels of bacterial transcripts (bacterial UMI ≤4) or are completely bacterial negative (bacterial UMI 0) (Fig. 3a). Additionally, we have observed that by increasing the bacterial UMI threshold, this will reduce the total number of cell-associated bacterial taxa, whereby many of them had bacterial UMI values of 1 (Fig. 3b and Supplementary Table 1). In our analysis, by increasing the bacterial UMI threshold to identify bacteria-associated cells for gene expression comparison against bacterial negative cells, we detected an increased number of altered host genes between these groups verses the number of altered genes when using a bacterial UMI 1 threshold (Fig. 3c and Supplementary Table 2). However, it is important to note that in this cell-type-independent analysis, many of the differentially expressed genes are reflective of the most dominant cell types and may mask the gene expression profiles from other unrepresented cell clusters or types from the same tumor tissue.

Moreover, we showed that host single cells can be assessed at the bacterial kingdom level (Total Bac.+ versus Total Bac.−) or at a specific taxa level such as the *Fusobacterium* genus, a dominant cell associated taxa in this OSCC single-cell sample (*Fuso.*+ versus Total Bac.−) (Fig. 3b,c). We observed variations in gene expression and pathway analysis at different bacterial taxonomic levels, probably due to specialized interactions between eukaryotic cells with specific bacterial taxa (*Fuso*.+ versus *Fuso*.−) (Fig. 3c,d and Supplementary Tables 2 and 3). It is important to note that single cells from tumor specimens at nonmucosal sites, or those that have been pretreated with antibiotics or chemotherapeutics that impact bacterial viability^6,35^, may have low numbers of bacteria-associated single cells and low bacterial UMI counts per single cell.

**Fig. 4 |:**
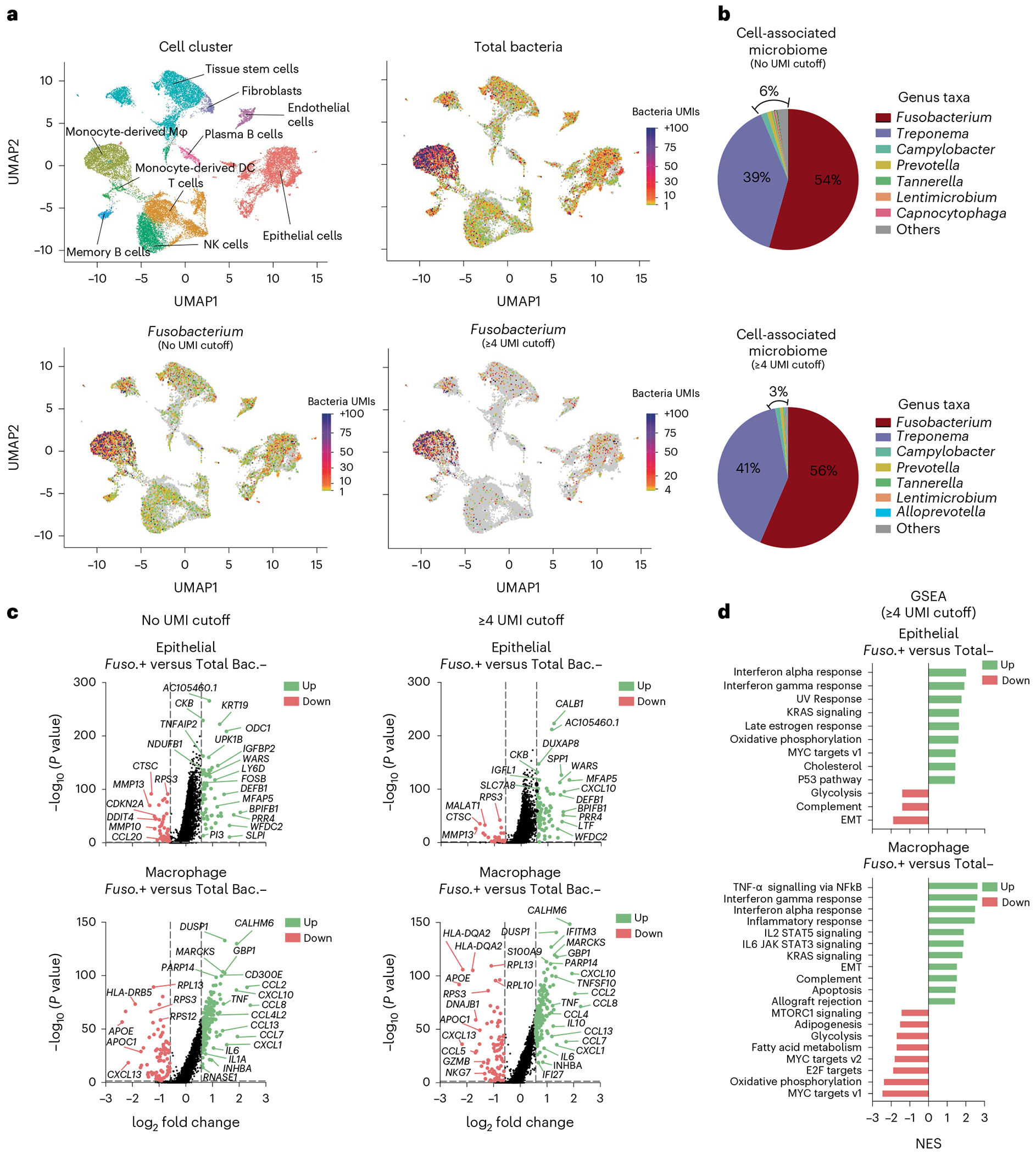
Single-cell RNAseq analysis in specific cell clusters from OSCC tumor samples containing a relatively high load of cell-associated bacteria (High_Bac cohort) following data integration. **a**, UMAP plots showing the cellular composition of the integrated data from the High_Bac cohort (*n* = 4 OSCC tumors) and the distribution of total bacteria or *Fusobacterium* with and without a UMI threshold as it is indicated. Color bars indicate the bacterial UMI transcriptional load. **b**, Pie charts of the cell-associated microbiome showing the most dominant bacterial genera in the single cells from the integrated data from the High_Bac cohort with and without a ≥4 UMI threshold as it is indicated. See also Supplementary Table 4. **c**, Volcano plots showing the differentially expressed genes in the macrophage and epithelial cell clusters when comparing *Fusobacterium*-positive single cells (*Fuso*.+) against total bacteria-negative single cells (Total Bac.−) with and without a UMI threshold of ≥4 as indicated. Dashed lines indicate the threshold of significant gene expression defined as the log_2_ fold change ≤−0.58 and ≥0.58 with a −log_10_ *P* value ≥1.301. Fold changes and *P* values were calculated by using a LMM, followed by a Benjamini-Hochberg multiple correction test. See also Supplementary Table 5. **d**, GSEA analysis showing the signaling pathways that are differentially regulated comparing *Fuso*.+ versus Total Bac.–single cells in the epithelial and macrophage cell cluster from the High_Bac cohort applying a UMI threshold of ≥4 for positive cells. A Wilcoxon rank sum test was implemented to calculate the normalized enrichment score. The data used to generate this figure have been published previously^8^.

To assess patient single-cell specimens with varying levels of cell-associated bacteria, we integrated tumor single-cell data from specimens with a relatively high cell-associated bacterial load (*n* = 4 OSCC specimens) and specimens with a lower cell associated bacteria load (*n* = 2 OSCC specimens). In the single cells from OSCC tumors with high cell-associated bacterial load (Fig. 4a), we again observed that the anaerobes *Fusobacterium* and *Treponema* are the dominant taxa (Fig. 4b and Supplementary Table 4). Additionally, bacterial transcripts are predominantly detected within the immune and epithelial cell clusters (Fig. 4a). Comparative analysis of single-cell types based on bacterial status at different taxonomic levels revealed an increased expression of proinflammatory genes in bacteria-associated single cells from the macrophage cell cluster (Fig. 4c,d and Supplementary Tables 5 and 6).

**Fig. 5 |:**
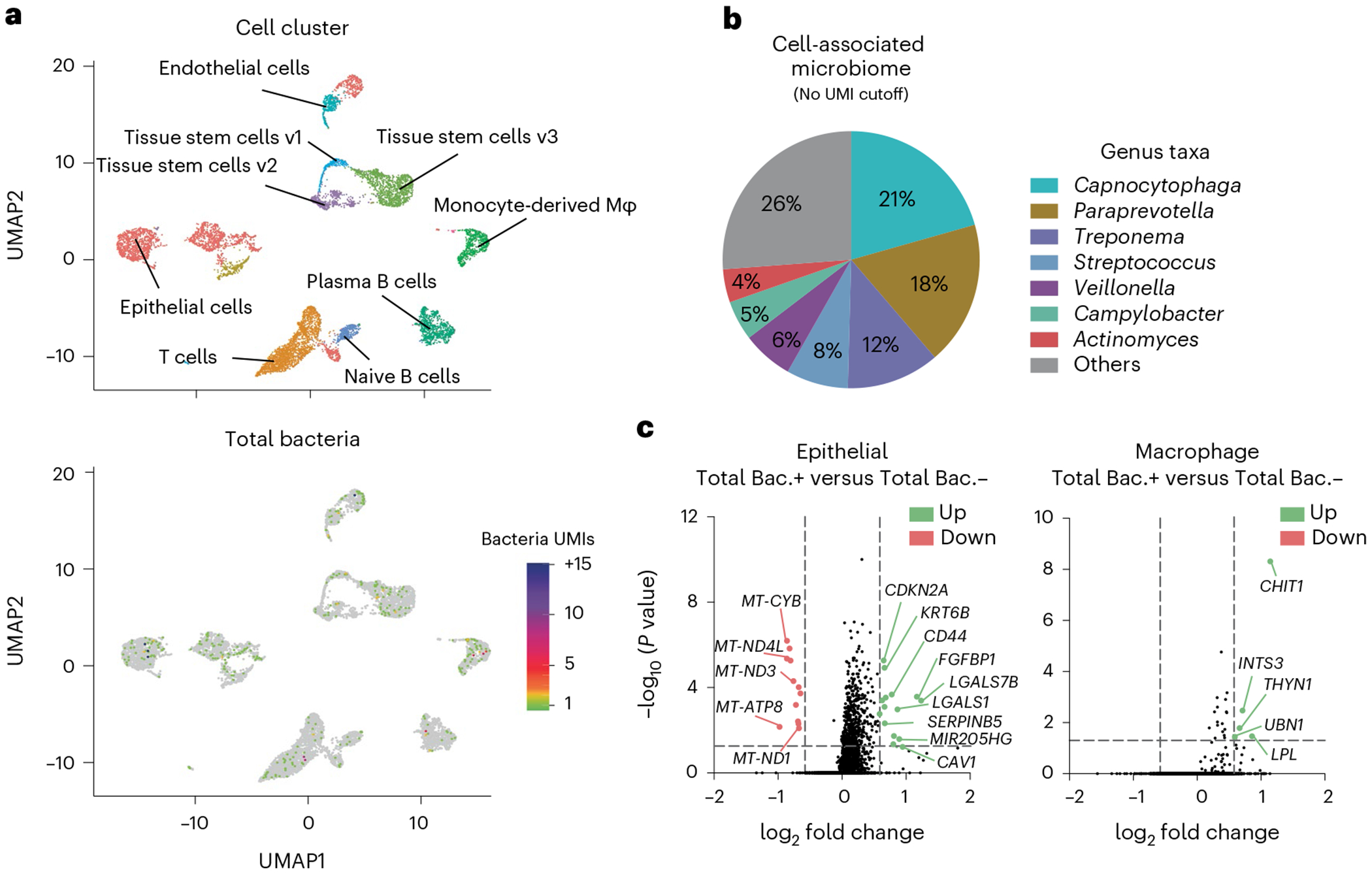
Single-cell RNAseq analysis in specific cell clusters from two OSCC tumor samples containing low bacteria load (Low_Bac) following data integration. **a**, UMAP plots showing the cellular composition and distribution of total bacteria in single cells from tumor samples with low cell-associated bacteria load (*n* = 2) after data integration. Color bar indicates the transcriptional UMI bacteria load. **b**, Pie chart of the cell-associated microbiome showing the most frequent bacterial genera detected with single cells from the Low_Bac cohort following data integration without applying any UMI cutoff. See also Supplementary Table 7. **c**, Volcano plots showing the genes that are differentially expressed when comparing total bacteria-positive cells (Total Bac.+) against total bacteria negative cells (Total Bac.−) epithelial or macrophage clusters as shown. Dashed lines indicate the threshold of significant gene expression defined as the log_2_ fold change ≤−0.58 and ≥0.58 with a −log_10_ *P* value ≥1.301. Fold changes and *P* values were calculated by using a LMM, followed by a Benjamini–Hochberg multiple correction test. Cells positive for *Patulibacter, Pseudomonas* and *Sphingomonas* were excluded from this analysis given the high likelihood that these bacterial taxa are contaminants introduced either from the environment or reagents and kits used. See also Supplementary Table 8. The data used to generate this figure have been published previously^8^.

In tumors containing relatively lower cell-associated bacterial load (Fig. 5a), we detected a range of bacterial genera (Fig. 5b and Supplementary Table 7). As noted earlier, samples with low bacterial loads are particularly sensitive to skew from contaminating bacterial taxa that may be present in the experimental kits or reagents used^29,34^. As with all microbiome studies, it is important to assess whether the bacterial taxa detected make biological sense in the context of the disease or tissue type. Many contaminating bacterial taxa may be environmental microbes not associated with human health or disease. For example, *Patulibacter, Pseudomonas* and *Sphingomonas* taxa were detected in this low bacterial biomass cohort but were not included for downstream analysis due to their high likelihood of being contaminants^27^. Additionally, in this cohort, low bacterial UMI levels were detected across a range of cell clusters and cell types (Fig. 5a). To identify genes impacted by cell-associated bacteria, we performed cell-type-specific analysis of single cells based on bacterial status (Fig. 5c,d). Unsurprisingly, the single-cell cohort with a relatively lower cell-associated bacterial load had less bacterial-dependent differential gene expression (Fig. 5c,d and Supplementary Table 8) compared with the single-cell cohort with high cell-associated bacterial load (Fig. 4c,d). This variation may be due to the difference in cell-associated bacterial taxa detected between the two cohorts or may be driven by the impact of bacterial load and prevalence within the single cells.

In summary, the INVADEseq approach will facilitate the identification and analysis of host–bacterial transcriptional interactions at the single-cell level from in vitro co-culture experiments, complex tissues and bodily fluids, across a range of health and disease states.

## Supplementary Material

Supplementary materials 1

Supplementary materials 2

3

## Figures and Tables

**Table 1 |:** Troubleshooting table

Step	Problem	Possible reason	Solution
1, 21, 148–149	Low viability of cells after tumor processing	Tumors can be damaged during the surgical resection. Additionally, some tumor tissues contain large areas of necrotic areas where tumor cells are more likely to experience cell death during tumor processing	It is advisable to process the tumor samples as soon as possible, optimizing the time from the resection of the tumor and the processing of such samples. To enrich for viable cells, it is recommended to use the dead cell removal protocol (Step 21). Furthermore, by using computational programs, low-quality cells (Seurat, Steps 148 and 149) can be removed from the analysis
4	Induction of transcriptomic artifacts due to tumor processing	During tumor processing it is possible to induce immediate-early genes associated with cellular stress masking the gene signatures associated with the biological agent in question	To avoid transcriptomic artifacts due to tumor processing the addition of 45 μM actinomycin D (for 35 °C) (Optional, Step 4) to the mix of enzymes during the digestion has been demonstrated to reduce transcriptional artifacts^33^
6, 21, 53	Low cDNA yields for GEX gene libraries	Low cDNA yields could be the result of low mRNA content of specific cell types (e.g., neutrophils). Inadequate cell counting can lead to loading fewer cells than recommended. Harsh conditions during tissue processing lowering the cell viability. Loss of cDNA content can be due to emulsion breaking and washing steps. cDNA overfragmentation can lead to reduce cDNA yields	If the samples are suspected to contain low mRNA yields it is recommended to change the program settings of the gentleMACS Octo Dissociator (37C_h_TDK_1 or 37C_h_TDK_2 for soft and medium tissues respectively), thus reducing tissue damage. It is also recommended to use automated cell counters (Countess II FL Automated Cell Counter) for accurate calculations of cell concentration. If the cDNA yields are low after tumor processing and single-cell acquisition it is recommended to increase by one or two PCR cycles during the amplification steps
21, 25-31	Reagent clogs during preparation of gel bead-in-emulsion	Clogs during preparation of gel bead-in-emulsions are generally caused by mishandling the gel beads or large clumps of cells or debris in the sample This could be caused by poor sterile conditions, clumps of gel bead or inadequate generation of single cells during sample preparation	To avoid gel bead clumps it is recommended to vortex the beads for 1 min and resuspend the solution by pipetting before use. It is also important to handle the gel beads in sterile environments. To reduce the frequency of clogs in the microfluidic system it is recommended to store the Chip K and chip holders in areas free of dust and debris. To ensure a single-cell suspension, it is recommended that the sample be passed through 70-μm cell strainers. It is also important to load an adequate concentration of cells, in the range between 200 and 700 cells/μl
32	Chromium Controller malfunctions	This is generally caused by an inadequate assembly (or loading) of the chip holder into the Chromium controller	If there are errors during single-cell acquisition, eject the tray from the controller and readjust the 10x chip holder. Ensure that the 10x gasket is properly install by aligning the holes with the wells from the Chip K. Place the chip holder assembly back into the tray of the controller and run the samples again. If the errors persist, contact technical support from 10x Genomics (support@10xgenomics.com)
38, 100, 111, 121	Low cDNA yields for 16S rRNA gene libraries	Loss of cDNA content can be due to emulsion breaking during the separation of the aqueous solution from the partitioning oil in Step 38. Washing steps can lead to lower cDNA yields. Additionally, narrow size selection of DNA fragments using the BluePippin platform could also lead to reduced yields	If the cDNA yields are low after the washing steps, it is recommended to increase by one or two PCR cycles during the amplification steps. In addition, broaden the collection range of the distribution size (bp) of cDNA fragments by using the BluePippin platform, thus increasing the overall DNA content. However, this could increase the likelihood of selecting undesired DNA fragments
			Increasing the number of reads per cell enhances the probability of capturing bacterial transcripts of low abundance since they are a minor component from the total human transcripts
139–145	Not detecting bacterial transcripts associated with single cells	The INVADEseq approach will only be successful if there are bacteria associated with mammalian cells. If bacteria are not associated with mammalian cells then bacterial transcripts will not be detected at the single-cell level	To maximize the successful application of the INVADEseq approach to a particular specimen or disease type with unknown microbial load, confirmation of intracellular or cell-associated bacteria via RNAscope imaging with a eubacterial probe is recommended^8^
160–162	Detecting unusual or unexpected bacterial taxa associated with single cells	It is important to determine whether the bacterial taxa detected in the specimen make biological sense. In general, tissue microbiota are considered to be low biomass and such specimens are more susceptible to microbiome skew from contamination^34^. Detection of taxa not typically associated with the host under analysis should raise caution	The INVADEseq computational pipeline assesses Read 1 for bacterial taxonomic assignment. Validation of the taxonomic assignment with Read 2 will increase stringency of taxa assignment. Applying in silico microbiome contamination prediction tools^29^ may help in distinguishing contaminants from true specimen associated reads

## Data Availability

Raw sequences data from INVADEseq bacterial 16S rRNA and human (GEX) gene libraries are available in the NCBI Sequence Read Archive repository under the Bioproject accession number PRJNA811533.

## Key points

- Invasion-adhesion-directed expression sequencing uses a modified 10x Genomics 5’ single-cell RNA sequencing protocol, introducing a primer targeting the bacterial 16S ribosomal RNA gene as well as the standard primer for eukaryotic poly(A) RNA selection to identify cell-associated bacteria and the host transcriptome.
- Invasion–adhesion-directed expression thus overcomes the limited ability of standard single-cell RNA sequencing approaches to capture the microbial component of the tumor microenvironment, facilitating analysis of host–bacterial interactions at the single-cell level.
