# Supplementary material for: INVADEseq to identify cell-adherent or invasive bacteria and the associated host transcriptome at single-cell-level resolution
Source: Nat Protoc. Author manuscript; Available in PMC 2024 Jan 16. (PMC10790651; doi:10.1038/s41596-023-00888-7)
Supplement: Supplementary materials 2 [file NIHMS1955151-supplement-Supplementary_materials_2.pdf]

nature protocols

https://doi.org/10.1038/s41596-023-00888-7

Protocol

Check for updates

INVADEseq to identify cell-adherent or invasive bacteria and the associated host transcriptome at single-cell-level resolution

In the format provided by the authors and unedited

nature protocols
https://doi.org/10.1038/s41596-023-00888-7

Protocol

Check for updates

INVADEseq to identify cell-adherent or invasive bacteria and the associated host transcriptome at single-cell-level resolution

In the format provided by the authors and unedited

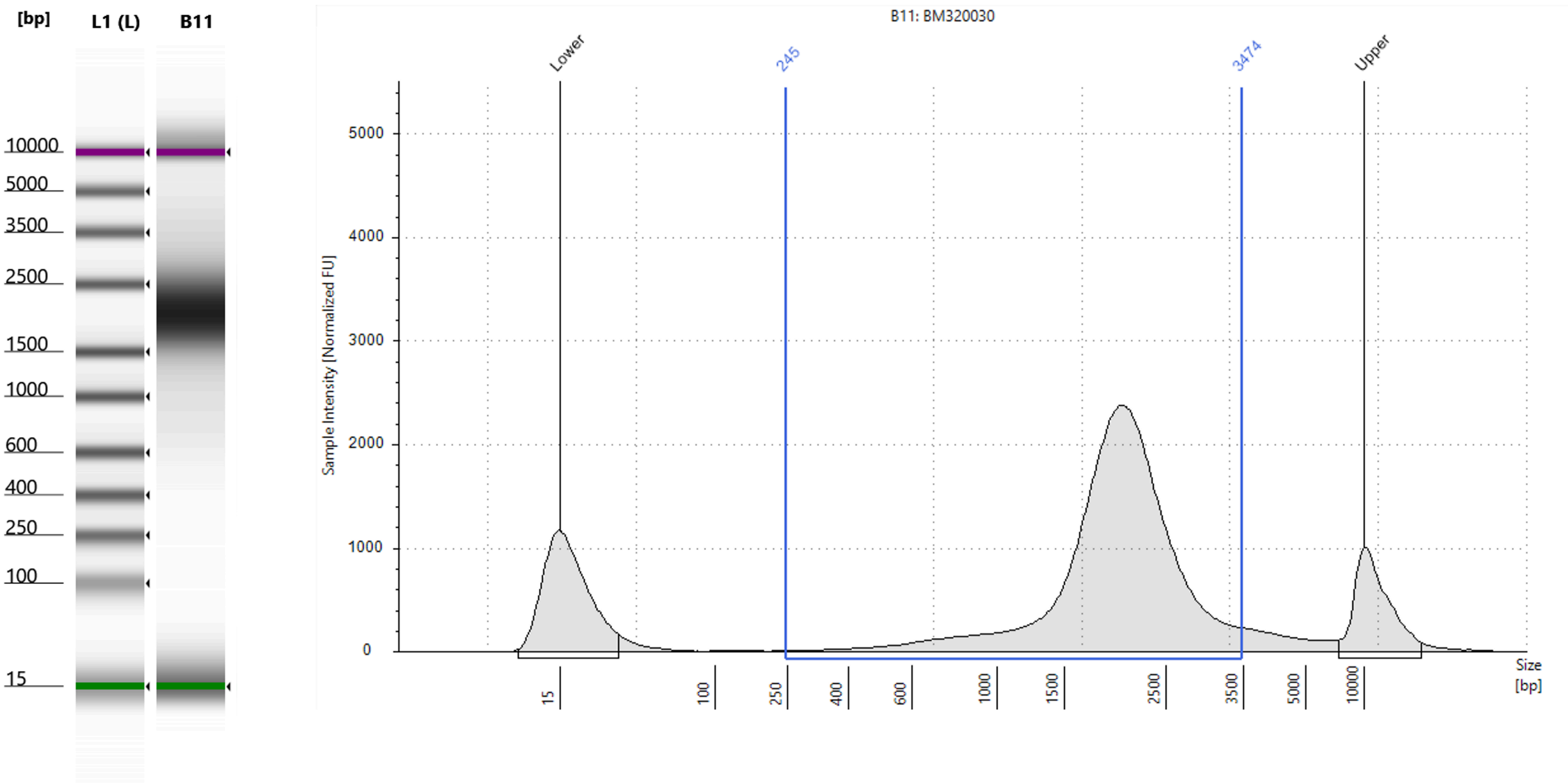

Supplementary Figure 1: Bacterial enrichment library size distributions.

Example of pseudo-gel image (left) and the corresponding trace (right) of a bacterial enrichment library from an oral tumor sample processed for INVADEseq.
